# Genetic Aberrations and Interaction of *NEK2* and *TP53* Accelerate Aggressiveness of Multiple Myeloma

**DOI:** 10.1002/advs.202104491

**Published:** 2022-01-27

**Authors:** Xiangling Feng, Jiaojiao Guo, Gang An, Yangbowen Wu, Zhenhao Liu, Bin Meng, Nihan He, Xinying Zhao, Shilian Chen, Yinghong Zhu, Jiliang Xia, Xin Li, Zhiyong Yu, Ruixuan Li, Guofeng Ren, Jihua Chen, Minghua Wu, Yanjuan He, Lugui Qiu, Jiaxi Zhou, Wen Zhou

**Affiliations:** ^1^ State Key Laboratory of Experimental Hematology Key Laboratory of Carcinogenesis and Cancer Invasion, Ministry of Education Key Laboratory of Carcinogenesis National Health and Family Planning Commission; Department of Hematology Xiangya Hospital Central South University Changsha Hunan 410028 China; ^2^ Cancer Research Institute School of Basic Medical Science Central South University 110 # Xiangya street Changsha Hunan 410028 China; ^3^ Xiang Ya School of Public Health Central South University Changsha Hunan 410028 China; ^4^ State Key Laboratory of Experimental Hematology Institute of Hematology & Blood Diseases Hospital Chinese Academy of Medical Science & Peking Union Medical College Tianjin 300041 China; ^5^ Shanghai Center for Bioinformation Technology Shanghai 201203 China; ^6^ The third Xiangya Hospital of Central South University Changsha Hunan 410013 China; ^7^ Department of Pathology Changsha eighth hospital Changsha Hunan 410199 China

**Keywords:** amplification, multiple myeloma, *NEK2*, *TP53*, transcriptional regulation

## Abstract

It has been previously shown that (never in mitosis gene A)‐related kinase 2 (*NEK2*) is upregulated in multiple myeloma (MM) and contributes to drug resistance. However, the mechanisms behind this upregulation remain poorly understood. In this study, it is found that amplification of *NEK2* and hypermethylation of distal CpG islands in its promoter correlate strongly with increased *NEK2* expression. Patients with *NEK2* amplification have a poor rate of survival and often exhibit *TP53* deletion, which is an independent prognostic factor in MM. This combination of *TP53* knockout and *NEK2* overexpression induces asymmetric mitosis, proliferation, drug resistance, and tumorigenic behaviors in MM in vitro and in vivo. In contrast, delivery of wild type p53 and suppression of NEK2 in *TP53^−/−^* MM cell lines inhibit tumor formation and enhance the effect of Bortezomib against MM. It is also discovered that inactivating p53 elevates *NEK2* expression genetically by inducing *NEK2* amplification, transcriptionally by increased activity of cell cycle‐related genes like E2F8 and epigenetically by upregulating DNA methyltransferases. Dual defects of *TP53* and *NEK2* may define patients with the poorest outcomes in MM with p53 inactivation, and NEK2 may serve as a novel therapeutic target in aggressive MM with p53 abnormalities.

## Graphical abstract

Patients with *NEK2* amplification are more prevalent in the *TP53*‐deleted subgroup of multiple myeloma (MM) and have a poor rate of survival. Dysfunctional p53 elevates *NEK2* expression genetically by inducing *NEK2* amplification, transcriptionally by mediating E2F8 activity, and epigenetically by increasing the expression of DNMTs. NEK2 may serve as a novel therapeutic target in aggressive MM with p53 abnormalities.

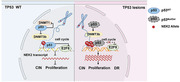

## 1 Introduction

Multiple myeloma(MM) is a cancer of terminally differentiated plasma cells and is the second most common hematological malignancy.^[^ ^1^ ^]^ The pathogenesis of MM involves several genetic alterations.^[^ ^2^ ^]^ These changes include primary cytogenetic abnormalities (e.g., translocations involving chromosome 14q and trisomies of odd‐numbered chromosomes) and secondary lesions (e.g., gain of chromosome 1q and loss of chromosome 17p).^[^ ^3^ ^]^

Cancer‐related genes are broadly grouped into oncogenes and tumor suppressor genes. The abnormal activation of oncogenes such as *CCND1*, *CCND2*, *CCND3*, and *FGFR3* has been reported in MM.^[^ ^2^, ^4^ ^]^ DNA gains and losses that result in copy number alterations cause oncogene activation and tumor suppressor gene inactivation; these are the driving events leading to the development and progression of MM.^[^ ^1^, ^2^, ^5^ ^]^ For example, the amplification of chromosome 1q, which harbors a number of potentially relevant oncogenes such as *CKS1B*,^[^ ^6^ ^]^ *ILF2*,^[^ ^7^ ^]^ *ANP32E*,^[^ ^8^ ^]^ and *PDZK1*,^[^ ^9^ ^]^ contributes to MM cell proliferation. Deletion of 17p, where *TP53* is located, also plays an important role in MM progression.^[^ ^10^ ^]^ However, how these oncogenes collaborate with tumor suppressor genes to accelerate MM initiation and progression is still poorly understood.

We have previously shown that increased chromosomal instability (CIN) signature is linked to drug resistance (DR) in MM.^[^ ^11^ ^]^ (Never in mitosis gene A)‐related kinase 2 (*NEK2*), a CIN gene located at 1q32.2, is the most significant. NEK2 is a serine/threonine kinase^[^ ^13^ ^]^ that induces tumor cell proliferation, metastasis and drug resistance through regulation of several oncogenes or cell cycle‐related molecules including AKT, *β*‐catenin and enhancer of zeste homolog 2 (EZH2)^[^ ^15^ ^]^ in MM and other types of cancer.^[^ ^11^, ^16^ ^]^ It is associated with poor outcomes and drug resistance due to activating efflux drug pumps,^[^ ^11^ ^]^ autophagy,^[^ ^14^ ^]^ and ALDH1A1 in MM.^[^ ^12^ ^]^ Although several critical substrates and molecules downstream of NEK2 are involved in tumorigenesis, the mechanisms by which *NEK2* is activated and cooperates with other molecules—especially key tumor suppressor genes like *TP53*—in MM cells are largely unknown.

Since 1q amplification and 17p deletion are both markers of the poorest prognosis in MM patients, we hypothesize that activated NEK2 and inactive p53 may have synergistic effects in MM progression. In this study, we determine the correlation of genetic aberrations and the functional relationship between *TP53* and *NEK2* in MM in vitro and in vivo.

## 2 Results

### 2.1 *NEK2* Amplification and Promoter Hypermethylation Correlate with its Upregulation and are Associated with Poor Prognosis in MM

**Figure 1:**
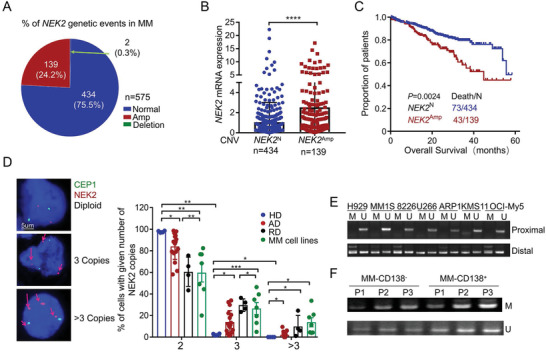
*NEK2* amplification in MM patients is associated with poor outcomes. A) Assessment of *NEK2* CNV distribution in MM patients (*n* = 575). B) The correlation between CNV and *NEK2* mRNA expression, and data presented as mean ± SD, *n* = 573, *p*‐values are calculated using unpaired t test, ****p < 0.0001. C) Kaplan‐Meier analysis of overall survival in MM patients with or without *NEK2* CNV amplification (*n* = 574). D) Left: Representative images of FISH analysis using probes specific for CEP1 (Green) and *NEK2* (Red) in eight MM cell lines, healthy donors (HD, *n* = 4), and newly‐diagnosed (AD, *n* = 17) and relapsed (RD, *n* = 4) MM patients. Upper: normal diploid. Middle: three copies. Lower: four copies (*NEK2* amplification). Right: scatter plot showing statistical results of the proportion of cells with different *NEK2* copy numbers in MM patients and MM cell lines. Data presented as mean ± SD, *p*‐values are calculated using unpaired t test, *p < 0.05, **p < 0.01, ***p < 0.001. The MSP results of *NEK2* proximal and distal CpG islands in E) seven MM cell lines and F) three MM patients. M, MSP products using methylation‐specific primers; U, MSP products using methylation‐unspecific primers.

Genetic lesions (including translocation, amplification and mutations) and epigenetic changes (e.g., DNA methylation, microRNA regulation, and transcriptional regulation) cause aberrant gene expression.^[^ ^17^ ^]^ To investigate the mechanisms underlying aberrant expression of *NEK2* in MM, we first analyzed DNA copy number variations (CNVs) and expression of *NEK2* by exome‐sequencing and RNA‐sequencing of 575 primary MM samples from the Multiple Myeloma Research Foundation (MMRF) CoMMpass database. We found that 24.2% (139/575) of the patients showed *NEK2* amplification (Amp) (**Figure** **1**A), which correlated strongly with elevated *NEK2* expression (Figure 1B). Moreover, a Kaplan‐Meier survival analysis of the CNVs database containing 573 MM patients revealed that patients with *NEK2* amplification had a significantly shorter overall survival (OS) (*NEK2^Amp^* group, median, 40 months) than the patients with a normal copy number (*NEK2^N^* group, median, 60 months, Figure 1C). Subsequent fluorescence in‐situ hybridization (FISH) experiments using a probe for *NEK2* and chromosome 1 control (CEP1) further confirmed these findings (Figure 1D). Compared with healthy donors (HD), *NEK2* amplification (defined as ≥20% of CD138^+^ cells with three or more FISH signals) was detected in 23.5% (5/17), 75% (3/4), and 87.5% (7/8) of newly diagnosed MM patients (AD), relapsed diagnosed MM patients (RD) and MM cell lines, respectively (Figure 1D and Table S1, Supporting Information), suggesting a link between *NEK2* amplification and poor prognosis in MM.

Next, to determine the status of *NEK2* mutations in MM cell lines, we analyzed all *NEK2* exons and its promoter region from −1018 to +1 bp in eight MM cell lines. One point mutation (A→G, N354S) of seven exons was found only in the U266 cell line (Figure S1A,B, Table S2, Supporting Information), suggesting that the mutation may not be responsible for upregulation of *NEK2* mRNA. Furthermore, two CpG islands in the regions from −700 to −500 bp (distal) and −200 to +1 bp (proximal) were observed in the *NEK2* promoter region (Figure S1B, Supporting Information). Methylation‐specific PCR (MSP) revealed unmethylation of the proximal CpG island and partial methylation of the distal CpG island in seven MM cell lines and three MM patients (Figure 1E,F). The sequencing of MSP products further showed that all the cytosine residues were converted to thymine except for those in methylated CpG dinucleotides, indicating the presence of methylated cytosines in these CpG dinucleotides (Figure S1C, Supporting Information). These results suggest that amplification and distal methylation of *NEK2* might be associated with its aberrant overexpression in MM.

### 2.2 *NEK2* Amplification Correlates with Deletion/Mutation of *TP53* in MM

**Figure 2:**
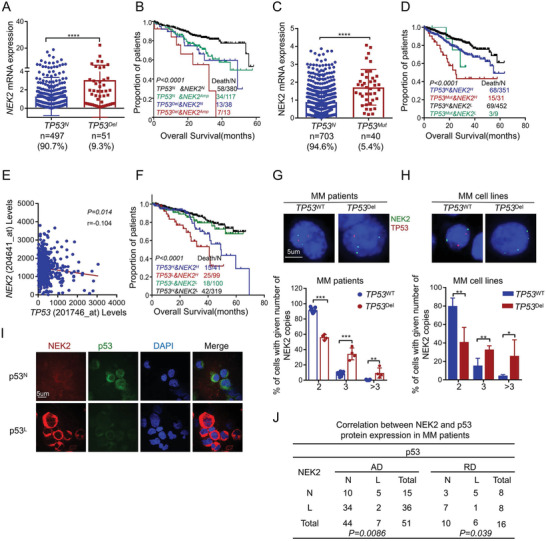
*NEK2* expression is elevated in MM patients with *TP53* lesions. A) *NEK2* mRNA levels in MM patients with (*n* = 51) or without (*n* = 497) *TP53* deletion, and data presented as mean ± SD, *p*‐values are calculated using unpaired t test, ****p < 0.0001. B) Kaplan‐Meier analyses of overall survival in MM patients with *TP53^N^* & *NEK2^N^* (normal), *TP53^N^* & *NEK2^Amp^* (Amplification), *TP53^D^* (deletion) &*NEK2^N^*, and TP53^D^ & NEK2^Amp^ (*n* = 548). C) *NEK2* mRNA levels in MM patients with (*n* = 40) or without (*n* = 703) *TP53* mutation, and data presented as mean ± SD, *p*‐values are calculated using unpaired t test, ****p < 0.0001. D) Kaplan‐Meier analyses of overall survival in MM patients with wild type *TP53* (*TP53* ^N^) & low expression *NEK2* (*NEK2^L^*), *TP53^N^* & high expression *NEK2* (*NEK2^H^*), mutant *TP53* (*TP53^mut^*) & *NEK2^L^* and *TP53^mut^* & *NEK2^H^* (*n* = 743). E) Correlation between *NEK2* and *TP53* mRNA expression in the TT2 and TT3 trial form GEP data (GSE2658, *n* = 559, *p*‐values are calculated Pearson correlation coefficient ). F) Kaplan‐Meier analyses of overall survival in TT2 and TT3 MM patients (*n* = 559) with high *NEK2* (*NEK2^H^*) & low *TP53* expression (*TP53^L^*), *NEK2^H^* & high *TP53* expression (*TP53^H^*), low *NEK2* (*NEK2^L^*) & *TP53^L^* and *NEK2^L^* and *TP53^H^*. G) Scatter plot showing the percentage of cells with amplified *NEK2* copy number in MM patients with or without *TP53* deletion. Data presented as mean ± SD, *p*‐values are calculated using unpaired t test, **p < 0.01, ***p < 0.001. H) Scatter plot showing the percentage of cells with amplified *NEK2* copy number in *TP53 ^WT^* or *TP53^−/−^* MM cells. Data presented as mean ± SD, *p*‐values are calculated using unpaired t test, *p < 0.05, **p < 0.01. I,J) Representative immunofluorescence images and statistical analysis for p53 (Green) and NEK2 (Red) protein expression in MM newly diagnosed patients (AD, *n* = 51) and relapsed patients (RD, *n* = 16, *p*‐values are calculated using one‐way ANOVA with Bonferroni correction).

It was previously shown that loss of *TP53* is an independent prognostic factor in MM^[^ ^10^ ^c]^ and that p53 can bind to the promoter of *NEK2*.^[^ ^18^ ^]^ These findings led us to assess the potential association between *TP53* and *NEK2* by using the CNVs database, containing 548 MM patients (excluding 25 MM patients with *TP53* amplification and two patients with *NEK2* deletion), from the MMRF CoMMpass database. First, we divided the samples into quartiles based on their expression levels of *TP53* (high = *TP53^H^*, low = *TP53^L^*) and *NEK2* (high = *NEK2^H^*, low = *NEK2^L^*). *TP53* deletion (*TP53^Del^*) was observed in 9.3% (51/548) of MM samples and contributed to aberrantly high expression of *NEK2* (**Figure** **2**A). Among the *TP53* ^Del^ MM patients, the frequency of *NEK2* amplification (*NEK2^Amp^*) was ~25.5% (13/51) and was also linked to high expression of *NEK2* (Figure S2A, Supporting Information). In addition, the proportion of patients with *NEK2* amplification increased in patients with *TP53* deletion (Figure S2B, Supporting Information). The analysis of the CNVs database further showed that patients with concomitant *TP53* deletion and *NEK2* amplification (*TP53^Del^&NEK2^Amp^*) had a significantly shorter OS (median, 32.4 months) than those in the *TP53^Del^* (49 months), *NEK2^Amp^* (44.8 months) and no *TP53^Del^* and *NEK2^Amp^* (*TP53^N^* &*NEK2^N^*) groups (not reached) (Figure 2B). Similarly, patients with concomitant *TP53^Del^* and *NEK2^H^* had a significantly shorter OS (median, 25.4 months) than others (*TP53^N^* & *NEK2^L^* and *TP53^Del^* & *NEK2^L^*, not reached; *TP53^N^* & *NEK2^H^*, 55.7 months; Figure S2C, Supporting Information). We also found that mutation of *TP53* (*TP53* ^Mut^) was observed in 5.4% (40/743) of MM samples and was associated with aberrantly high expression of *NEK2* (Figure 2C) using the MMRF CoMMpass database of 743 cases. The results further showed that patients with both *TP53* ^Mut^ and *NEK2^H^* had a significantly shorter OS (median, 25 months) than those without the two defects simultaneously (*TP53^N^* & *NEK2^L^* and *TP53^Mut^* & *NEK2^L^*, not reached; *TP53^N^* & *NEK2^H^*, 54.8 months; Figure 2D, Supporting Information 1).

To further examine the relationship between *NEK2* and *TP53* in other cancer types, we analyzed the copy number and mRNA levels of *NEK2* in 24 different cancer types with *TP53* abnormalities based on The Cancer Genome Atlas (TCGA) database. The cancer types included adrenocortical carcinoma, bladder urothelial cancer, breast invasive cancer, esophageal carcinoma, glioblastoma multiforme, kidney renal papillary cell carcinoma, liver hepatocellular carcinoma (LIHC), lung adenocarcinoma (LUAD), pancreatic adenocarcinoma, sarcoma, skin cutaneous melanoma, and stomach adenocarcinoma. We found that *NEK2* expression was significantly upregulated in *TP53^Del^* or *TP53^Mut^* cancer samples compared to samples with normal *TP53* genetic status (Figure S2D, Supporting Information). The copy number of *NEK2* was also amplified and *NEK2* mRNA levels were increased in *TP53* ^Del^ samples from these cancer types, except for LIHC and LUAD (Figure S2E, Supporting Information). These results implied that *TP53* deletion or mutation (*TP53* ^Del/Mut^) correlates with *NEK2* amplification and elevates *NEK2* expression in MM and other cancer types.

To assess the effect of *NEK2* and *TP53* expression and their roles in prognosis, we applied the Gene Expression Programming (GEP) database of 559 MM samples (GSE2658^[^ ^5^ ^]^). As shown in Figure 2E, *TP53* expression (low in MM) correlated inversely with *NEK2* expression in MM patients. Additionally, the MM patients with *TP53^L^* and concomitant *NEK2^H^* expression had a significantly inferior OS (median, 40.8 months), while patients with either *NEK2* ^L^ or *TP53^H^* had better outcomes (*TP53^H^* & *NEK2^L^* and *TP53^L^* & *NEK2^L^*, not reached; *TP53^H^* & *NEK2^H^*, 49 months; Figure 2F).

To further confirm whether *NEK2* was amplified in *TP53^Del^* patients, we performed FISH using probes targeting the *TP53* and *NEK2* gene loci in MM clinical samples. The results showed ≥20% of CD138^+^ cells with three or more signals marked as *NEK2* loci in 100% (4/4) of *TP53^Del^* MM samples (P31‐34, Table S1, Supporting Information), but not in seven *TP53* ^WT^ samples (P25‐30, Table S1, Supporting Information) (Figure 2G). We also found that the number of cells containing three copies of *NEK2* was significantly greater in *TP53^Del^* MM cell lines (including ARP1 and KMS11) than in *TP53* ^WT^ MM cell lines (including MM.1S and H929) (Figure 2H).

Finally, to verify whether the loss of p53 is linked to elevated NEK2 expression, we performed immunofluorescence (IF) to examine p53 and NEK2 protein levels in 51 newly diagnosed and 16 relapsed MM patients. As shown in Figure 2I,J, 9.8% (5/51) of newly diagnosed patients and 31.3% (5/16) of relapsed patients exhibited low p53 protein expression and high NEK2 protein expression, while in 66.7% (34/51) of new patients and 43.8% (7/16) of relapsed patients, the reverse was true. Experiments with Fisher's exact probability test implied that p53 protein expression correlated inversely with NEK2 protein expression in MM. These results suggested that patients of the *TP53^Del/Mut^* MM subgroup are prone to *NEK2* amplification and upregulation, leading to poor prognosis.

### 2.3 *TP53* Deletion/Mutation Upregulates *NEK2* in Myeloma Cells

**Figure 3:**
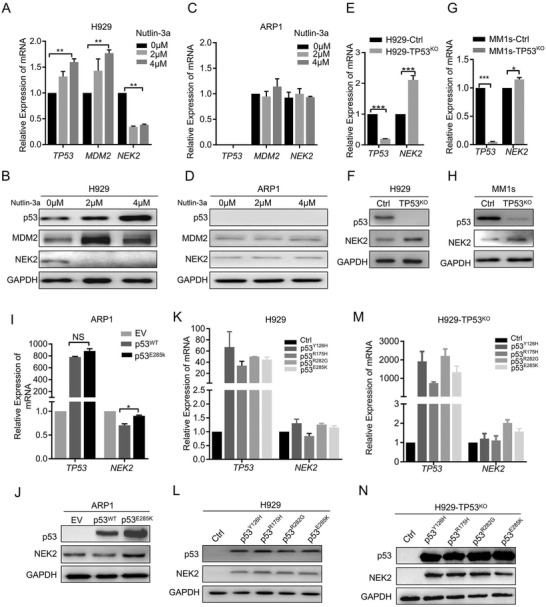
*NEK2* expression is reduced and correlates inversely with loss of wild type *TP53*in MM cells. A) Relative mRNA levels of *NEK2* and *TP53* in the H929 *TP53^WT^* MM cell line were detected with qPCR after treatment with nutlin‐3a for 48 h at concentrations of 0, 2, and 4 µM. Data presented as mean ± SD, *p*‐values are calculated using unpaired t test, *n* = 3, **p < 0.01. B) Relative protein levels of p53, NEK2, and GAPDH in *TP53^WT^* H929 cells after treatment with 0, 2, and 4 µM of nutlin‐3a for 48 h, as determined with immunoblotting. C) Relative mRNA levels of *NEK2* and *TP53* in the ARP1 *TP53^−/−^* MM cell line were detected with qPCR after treatment with nutlin‐3a for 48 h at concentrations of 0, 2, and 4 µM. D) Relative protein levels of p53, NEK2, and GAPDH in *TP53^−/−^* ARP1 cells after treatment with 0, 2, and 4 µM of nutlin‐3a for 48 h, as determined with immunoblotting. E) The mRNA levels of *TP53* and *NEK2* in H929 cells with or without *TP53* deletion edited by CRISPR/Cas9, as determined with qPCR. Data presented as mean ± SD, *p*‐values are calculated using unpaired t test, *n* = 3, ***p < 0.001. F) p53 and NEK2 protein levels in H929 cells with or without CRISPR‐Cas9‐mediated *TP53* deletion, as determined with immunoblotting. G) *TP53* and *NEK2* mRNA levels in MM.1s cells with or without CRISPR‐Cas9‐mediated *TP53* deletion, as determined with qPCR. Data presented as mean ± SD, *p*‐values are calculated using unpaired t test, *n* = 3, *p < 0.05, ***p < 0.001. H) The protein levels of p53 and NEK2 in MM.1s cells with or without CRISPR‐Cas9‐mediated *TP53* deletion, as determined with immunoblotting. I) Relative mRNA levels of *TP53* and *NEK2* in ARP1 cells were detected with qPCR 72 h after infection with virus containing WT or mutant *TP53* (E285K)‐expressing vectors. Data presented as mean ± SD, *p*‐values are calculated using unpaired t test, *n* = 3, *p < 0.05. J) Relative protein levels of p53, NEK2, and GAPDH in ARP1 cells were detected with immunoblotting 72 h after infection with virus containing WT or mutant *TP53* (E285K)‐expressing vectors. K) The mRNA levels of *TP53* and *NEK2* in H929 cells with or without *TP53* mutation, as determined with qPCR. L) The protein levels of p53 and NEK2 in H929 cells with or without *TP53* mutation, as determined with immunoblotting. M) The mRNA levels of *TP53* and *NEK2* in *TP53*‐deleted H929 (H929‐TP53^KO^) cells with or without *TP53* mutation, as determined with qPCR. N) The protein levels of p53 and NEK2 in *TP53*‐deleted H929 (H929‐TP53^KO^) cells with or without *TP53* mutation, as determined with immunoblotting.

To establish a causal relationship between *TP5*3 deletion/mutation and *NEK2* expression in MM, we induced p53 expression in H929 and ARP1 cell lines using different concentrations of nutlin‐3a. A dose‐dependent decrease of *NEK2* mRNA and protein levels and a dose‐dependent increase of *TP53* and *MDM2* mRNA and protein levels were observed in H929 cells (**Figure** **3**A,B) but not in *TP53^−/−^* ARP1 cells (Figure 3C,D).

We also assessed the effect of knocking out (KO) *TP53* in HEK293 cells using the CRISPR‐Cas9 gene editing technology. Cells infected with virus containing CRISPR‐Cas9 or CRISPR‐Cas9‐TP53 vector were successfully established and were dubbed HEK293‐Ctrl and HEK293‐TP53^KO^, respectively (Figure S3A–C, Supporting Information). *TP53* deletion in HEK293 cells (HEK293‐TP53^KO^) caused *NEK2* mRNA and protein levels to increase dramatically when compared with the control cells (Figure S3B,C, Supporting Information). Subsequently, p53 was successfully knocked out in H929 cells and knocked down in MM.1s cells established using the CRISPR‐Cas9 system. These p53‐deficient cell lines were named H929‐TP53^KO^ and MM.1s‐TP53^KO^, respectively. We observed increased *NEK2* mRNA and protein levels in both H929‐TP53^KO^ cells (Figure 3E,F) and MM.1s‐TP53^KO^ cells (Figure 3G,H).

We then obtained cell clones from H929‐TP53^KO^ cells via sequential dilution and found that the *NEK2* mRNA and protein levels were upregulated in clones with *TP53* deletion (C1, C2, and C4) and more strongly in those with *TP53* mutation (C3, C5) (Figure S3D,E, Supporting Information). The sequence forms of the DNA and protein from these clones were confirmed by sequencing (Figure S3F and Additional File S6, Supporting Information). Similar results were also seen in HCT116‐TP53^−/−^ cells (Figure S3G,H, Supporting Information) and the Trp53^tm1Tyj^ TP53‐knockout mouse genetic model (Figure S3I,J, Supporting Information).

In contrast, ectopic expression of WT *TP53* in ARP1 cells caused *NEK2* mRNA and protein levels to markedly decrease compared with empty vector (ARP1‐EV), while ectopic expression of mutant *TP53* with a hotspot mutation at *E285K* exerted no inhibitory effect (Figure 3I,J). Moreover, ectopic expression of mutant *TP53* with hotspot mutations observed in MM and other tumors (*Y126H*, *R175H*, *R282G*, and *E285K*) in H929‐TP53^WT^ and H929‐TP53^KO^ cells caused slight elevation of *NEK2* mRNA levels, while protein levels were increased dramatically (Figure 3K–N). These results indicate that *TP53* deletion or mutation induces *NEK2* upregulation.

### 2.4 NEK2 Activation and p53 Suppression Promote Mitotic Aberrations, Cell Proliferation, and Tumorigenesis in MM

To test the effect of dual defects of *NEK2* and *TP53* on MM, we overexpressed NEK2 in H929 cells (H929‐NEK2 OE) and H929‐TP53^KO^ cells (H929‐TP53^KO^/NEK2 OE), then performed functional assays in H929‐Ctrl, H929‐NEK2 OE, H929‐TP53^KO^, and H929‐TP53^KO^/NEK2 OE cells after determination of *TP53* and *NEK2* mRNA expression and protein levels (Figure S4A,B, Supporting Information).

**Figure 4:**
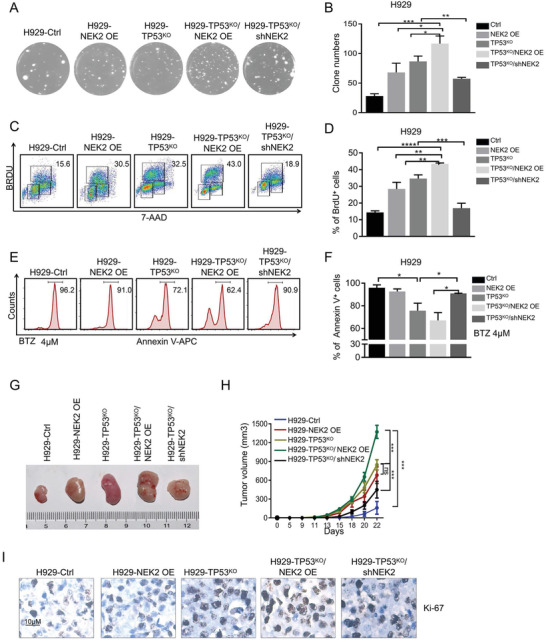
Dual defects in NEK2 and p53 enhance mitotic abnormalities, cell proliferation, apoptosis, and tumorigenesis in MM. A) Representative images of clonogenic analysis in H929‐Ctrl, H929‐TP53^KO^, H929‐NEK2 OE, H929‐TP53^KO^/NEK2 OE, and H929‐TP53^KO^/shNEK2 cells (images are shown in 4x magnification). B) Statistical analysis of clone numbers formed in soft agarose of the five cell groups shown in panel (A). Data presented as mean ± SD, *p*‐values are calculated using unpaired one‐way ANOVA with Dunnett post‐hoc test, *n* = 3, *p < 0.05, **p < 0.01, ***p < 0.001. C) Representative flow cytometry dot plots for detection of BrdU‐positive cells among H929‐Ctrl, H929‐TP53^KO^, H929‐NEK2 OE, H929‐TP53^KO^/NEK2 OE, and H929‐TP53^KO^/shNEK2 cells. D) Statistical analysis of the number of BrdU‐positive cells among the five cell groups shown in panel (C). Data presented as mean ± SD, *p*‐values are calculated using one‐way ANOVA with Dunnett post‐hoc test, *n* = 3, *p < 0.05, **p < 0.01, ***p < 0.001. E) Representative histograms for detection of apoptotic cells in H929‐Ctrl, H929‐TP53^KO^, H929‐NEK2 OE, H929‐TP53^KO^/NEK2 OE, and H929‐TP53^KO^/shNEK2 cells treated with 4 nM BTZ for 48 h. F) Statistical analysis of the percentage of apoptotic cells among the five cell groups shown in panel (E) after treatment with 4 nM BTZ for 48 h. Data presented as mean ± SD, *p*‐values are calculated using one‐way ANOVA with Dunnett post‐hoc test, *n* = 3, *p < 0.05, **p < 0.01, ***p < 0.001. G) Representative images of tumor xenografts from B‐NDG mice with subcutaneous injections of H929‐Ctrl, H929‐TP53^KO^, H929‐NEK2 OE, H929‐TP53^KO^/NEK2 OE, or H929‐TP53^KO^/shNEK2 cells into the right abdomen (6 mice measured for each group). H) Statistical analysis of tumor volumes of xenografts from B‐NDG mice as shown in panel (G) (6 mice measured for each group). Data presented as mean ± SD, *p*‐values are calculated using two‐way ANOVA with Dunnett post‐hoc test, *n* = 6, *p < 0.05, **p < 0.01, ***p < 0.001. I) Representative images for IHC detection of Ki‐67 protein in the tumor nodules derived from B‐NDG mice injected subcutaneously with H929‐Ctrl, H929‐TP53^KO^, H929‐NEK2 OE, H929‐TP53^KO^/NEK2 OE, or H929‐TP53^KO^/shNEK2 cells.

We first examined the role of NEK2 in cell growth by using the clonogenic soft agar assay and BrdU intake assay, finding that H929‐TP53^KO^/NEK2 OE cells showed a significant elevation in colony formation (117 ± 7) compared with other groups including H929‐NEK2 OE (68 ± 9), H929‐TP53^KO^ (87 ± 5), and H929‐Ctrl (28 ± 2, **Figure** **4**A,B). The percentage of BrdU‐positive cells in the H929‐TP53^KO^/NEK2 OE group (43.5 ± 0.4%) was also much higher than that for other groups including H929‐NEK2 OE (28.4 ± 2.0%), H929‐TP53^KO^ (34.7%), and H929‐Ctrl cells (14.3 ± 0.5%, Figure 4C,D). In addition, after NEK2 was depleted in H929‐TP53^KO^/shNEK2 cells via tetracycline‐inducible shRNA‐mediated knockdown, the number of colonies (57 ± 1, Figure 4A,B) and the percentage of BrdU‐positive cells (16.8 ± 1.7%, Figure 4C,D) were drastically reduced compared to those of H929‐TP53^KO^ cells.

Subsequently, we used an apoptosis assay to test the sensitivity of cells with combined *NEK2*/*TP53* defects to Bortezomib (BTZ, a proteasome inhibitor and a first‐line treatment in MM). We found that the percentage of apoptotic cells decreased significantly in the H929‐TP53^KO^ group (75.7 ± 3.3%), and more prominently in the H929‐TP53^KO^/NEK2 OE group (67.2 ± 4.8%), after BTZ treatment (4 or 2 nM) when compared with the H929‐Ctrl group (96 ± 1.8%) (Figure 4E,F, Figure S4C, Supporting Information). In addition, after NEK2 was depleted in H929‐TP53^KO^/shNEK2 cells, the percentage of apoptotic cells (91 ± 0.1%) was markedly increased compared with that in H929‐TP53^KO^ cells (Figure 4E,F, Figure S4C, Supporting Information).

Finally, we evaluated the effects of combined *NEK2* and *TP53* defects in vivo using MM xenograft models. We found that NEK2 overexpression and p53 knockdown separately could increase tumor growth, and this effect was significantly enhanced when the defects were combined in the H929‐TP53^KO^/NEK2 OE group; meanwhile, tumor growth was decreased in the H929‐TP53^KO^/shNEK2 group (Figure 4G,H, Figure S4D, Supporting Information). Immunohistochemistry (IHC) confirmed that the tumor cells from xenograft nodules were CD138^+^ plasma cells with NEK2 overexpression and p53 depletion (Figure S4E, Supporting Information). Also, the number of proliferative (Ki‐67^+^) cells increased significantly in the H929‐TP53^KO^/NEK2 OE group while decreasing in the H929‐TP53^KO^/shNEK2 group (Figure 4I, Figure S4F, Supporting Information). Thus, dual defects in *NEK2* and *TP53* promote proliferation and tumorigenesis in MM.

### 2.5 *TP53* Deletion Upregulates *NEK2* via the Regulation of Chromosomal Instability‐Related Genes

**Figure 5:**
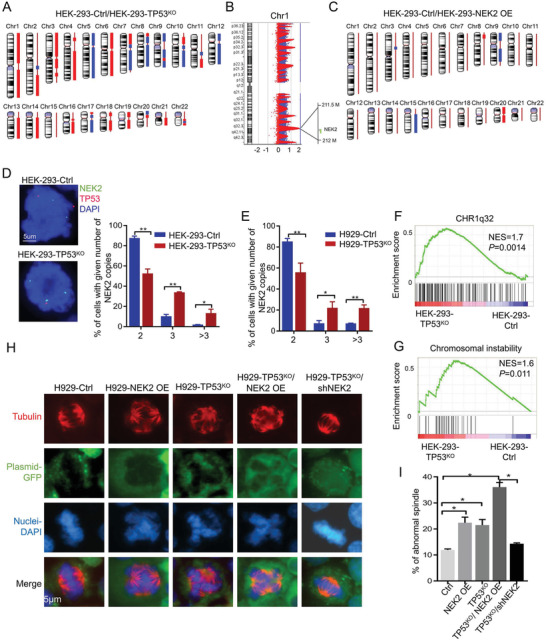
*TP53* deletion upregulates *NEK2* expression by inducing amplification and chromosomal instability in MM cells. A) Comparative analysis of HEK293‐TP53^KO^ versus HEK293‐Ctrl cells using CGH array. Red column represents gains of chromosomes. Blue column represents deletions. Chromosomal aberrations are across the whole genome. B) Schematic depiction of *NEK2* location in chromosome 1q21.1‐q44. C) Comparative analysis of HEK293‐NEK2 OE versus HEK293‐Ctrl cells using CGH array. Red column represents gain. Blue column represents deletion. D) Left, representative images of FISH analysis using probes targeting *TP53* (17P, Red) and *NEK2* (1q32.2, Green) in HEK293‐Ctrl and HEK293‐TP53^KO^ cells. Right, scatter plot showing the percentage of cells from both groups with amplified *NEK2* copy numbers. Data presented as mean ± SD, *p*‐values are calculated using unpaired t test, *n* = 3, *p < 0.05, **p < 0.01. E) Scatter plot showing the percentage of H929‐Ctrl and H929‐TP53^KO^ cells with amplified *NEK2* copy numbers as determined by FISH assay. Data presented as mean ± SD, *p*‐values are calculated using unpaired t test, *n* = 3, *p < 0.05, **p < 0.01. F,G) GSEA of enrichment, including chromosome 1q32 and chromosomal instability, from differentially expressed genes between HEK293‐Ctrl and HEK293‐TP53^KO^ cells. H) Representative images of spindles (Red: Tubulin), Plasmid‐GFP (Green), and nuclei (Blue) in H929‐Ctrl, H929‐TP53^KO^, H929‐NEK2 OE, H929‐TP53^KO^/NEK2 OE, and H929‐TP53^KO^/shNEK2 cells. I) Quantification showing the percentage of cells with abnormal monopolarity among each of the five groups shown in panel (H). All results are shown as means ± SD of three independent experiments, and at least 60 spindles per experiment were randomly chosen and counted in each experiment.Data presented as mean ± SD, p‐values are calculated usingone‐way ANOVA with Dunnett post‐hoc test, *p < 0.05.

Because of the complexity of genetic abnormalities in cancer, we used HEK293 cells, with their relatively normal phenotype and genetic background, to examine whether p53 deletion or NEK2 overexpression can induce CIN. A comparative genomic hybridization (CGH) array was performed to scan the entire genome of normal HEK293 cells (HEK293‐Ctrl), cells with NEK2 overexpression (HEK293‐NEK2 OE) and HEK293‐TP53^KO^ cells. Notably, compared with HEK293‐Ctrl, several high‐risk genetic abnormalities, including deletion of chromosomes 5, 7q, 8, 9, and 17p and some gains of chromosomes 1, 2q, 3p, 4p, 6, 13q, and 14q, were observed in HEK293‐TP53^KO^ cells (**Figure** **5**A, Table S3, Supporting Information). Fewer chromosomal changes were observed in HEK293‐NEK2 OE cells compared with HEK293‐Ctrl cells (Figure 5C, Table S4, Supporting Information). Interestingly, chromosome 1q21‐44, where NEK2 is located, was dramatically amplified in HEK293‐TP53^KO^ cells (Figure 5B).

To verify the findings from the CGH array, we subsequently performed FISH in HEK293‐Ctrl and HEK293‐TP53^KO^ cells using probes targeting the *NEK2* and *TP53* DNA regions. The number of cells with two copies of *NEK2* decreased, while the number of cells with three or more copies increased significantly in the HEK293‐TP53^KO^ cells compared with the HEK293‐Ctrl cells (Figure 5D). A similar pattern was observed for H929‐TP53^KO^ cells (Figure 5E). This evidence further suggests that p53 deletion is associated with *NEK2* amplification in MM.

Interestingly, differentially expressed genes (Tables S5 and S6, Supporting Information) were mainly found in the cytoplasm and nucleus. They were mostly involved in spindle formation, chromosomal stability and gene transcription, as revealed by R pathway enrichment analysis (Table S7 and S8, Supporting Information) when we used RNA‐seq to examine the gene expression profiles in HEK293‐Ctrl, HEK293‐TP53^KO^, and HEK293‐TP53^KO^/NEK2 OE cells. In addition, gene set enrichment analysis showed 1q32 amplification and major types of gene signatures enriched in CIN, cell proliferation, and p53 pathway (Figure 5F,G, Figure S5A, Supporting Information). The expression level of CIN‐related genes was significantly altered in HEK293‐TP53^KO^ cells and more prominently in cells with combined defects in *NEK2* and *TP53* (Figure S5B, Supporting Information). We then verified the changes in the expression of CIN‐related genes, including *TP53*, *NEK2*, *BUBR1*, *HEC1*, *AURKA*, *AURKB*, and *MAD2L1*, by using qPCR in HEK293 and H929 cells with or without *TP53* deletion (Figure S5C,D, Supporting Information). Thus, p53 deletion leads to *NEK2* amplification or overexpression, likely through the alteration of CIN‐related genes.

Next, we used a spindle formation assay to demonstrate that the percentage of cells with abnormal mitosis (e.g., asymmetric spindle division, multipolar division, and nuclear condensation) increased significantly in the H929‐NEK2 OE (22.4 ± 1.5%) and H929‐TP53^KO^ groups (21.5 ± 1.5%) when compared to the H929‐Ctrl group (11.9 ± 0.3%), and an even greater increase was observed in the H929‐TP53^KO^/NEK2 OE group (36.1 ± 1.2%, Figure 5H,I). We also depleted NEK2 in H929 cells with p53 deletion by using shRNA‐mediated NEK2 knockdown (denoted as the H929‐TP53^KO^/shNEK2 group), which rescued aberrant defects in spindle formation (14.3 ± 0.2%, Figure 5H,I).

### 2.6 *TP53* Deletion Enhances *NEK2* Expression Upregulation of E2F8

**Figure 6:**
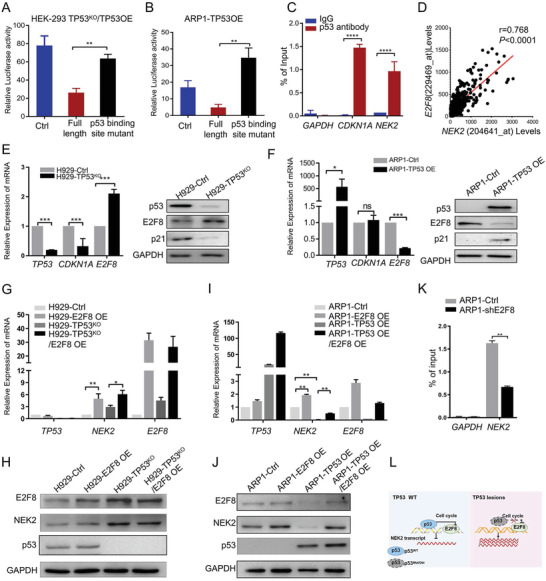
p53 deletion promotes aberrantly high *NEK2* expression through indirect transcriptional regulation. A,B) Luciferase activity driven by *NEK2* promoter with normal (full length) or mutant p53 binding site in HEK293‐TP53^KO^ and ARP1 cells transiently co‐transfected with p53 OE. Data presented as mean ± SD, *p*‐values are calculated using unpaired t test, *n* = 3, **p < 0.01. C) ChIP confirming that the transcription factor p53 specifically binds to the *NEK2* promoter region in H929 cells. Data presented as mean ± SD, *p*‐values are calculated using unpaired t test, *n* = 3, ****p < 0.0001. D) Correlation of expression between *NEK2* and *E2F8* in MM patients based on GEP database (GSE2658, *n* = 559, *p*‐values are calculated Spearman correlation coefficient ). E) The mRNA and protein levels of *TP53*, *CDKN1A* (*p21*), and *E2F8* in H929 cells with or without p53 deletion. Here and in panels (F–J), mRNA and protein data were obtained using qPCR and immunoblotting, respectively. Data presented as mean ± SD, *p*‐values are calculated using unpaired t test, *n* = 3, ***p < 0.001. F) The mRNA and protein levels of *TP53*, *CDKN1A* (*p21*), and *E2F8* in ARP1 cells with or without p53 overexpression. Data presented as mean ± SD, *p*‐values are calculated using unpaired t test, *n* = 3, *p < 0.05, ***p < 0.001. G,H) The mRNA and protein levels of *NEK2* and *E2F8* in H929‐Ctrl and H929‐TP53^KO^ cells with (H929‐TP53^KO^/E2F8 OE) or without E2F8 overexpression. Data presented as mean ± SD, *p*‐values are calculated using unpaired t test, *n* = 3, *p < 0.05, **p < 0.01. I,J) The mRNA and protein levels of *NEK2* and *E2F8* in ARP1‐EV and ARP1‐TP53 OE cells with (ARP1‐TP53 OE/E2F8 OE) or without E2F8 overexpression. Data presented as mean ± SD, *p*‐values are calculated using unpaired t test, *n* = 3, **p < 0.01. K) ChIP confirmed that the transcription factor E2F8 specifically binds to the *NEK2* promoter region. L) Schematic of p53 deletion enhancing *NEK2* expression through E2F8 upregulation.

A non‐canonical p53 recognition site (CGCCATGTTGGCCAGGCTGGTCT), identical to the site from the huntingtin gene promoter, was previously identified at the distal promoter region of *NEK2*.^[^ ^18^ ^]^ To test the function of this site, we constructed the full‐length *NEK2* promoter fragment (1098 bp from −1017 to −2 bp relative to the TSS, Figure S6A, Supporting Information) and a mutant *NEK2* promoter with deletion of the p53 binding site and inserted them into the luciferase reporter vector PGL3‐enhancer. We then co‐transfected the fusion constructs together with the *TP53* expression vector into HEK293‐TP53^KO^ and ARP1 cells. Luciferase activity in cells with the full‐length *NEK2* promoter sequence decreased dramatically upon p53 expression, while it was mostly unaffected in cells containing the mutant promoter with p53 binding site deletion (**Figure** **6**A,B, Figure S6B,C, Supporting Information). ChIP‐qPCR analysis with an anti‐p53 antibody showed enrichment of distal *NEK2* promoter sequences compared with the IgG isotype control, similar to the positive control *p21* gene (also known as cyclin dependent kinase inhibitor 1A, *CDKN1A*) (Figure 6C), indicating that p53 suppresses *NEK2* transcription by directly binding to the *NEK2* promoter.

Subsequently, we analyzed the differentially expressed genes (Table S9, Supporting Information) obtained from gene expression profiles in H929‐Ctrl, H929‐TP53^KO^, and H929‐TP53^KO^/NEK2 OE cells. CIN‐related genes, along with genes in the *cyclin* and *E2F* families, were differentially expressed in H929‐TP53^KO^ cells and, to an even larger degree, in H929‐TP53^KO^/NEK2 OE cells (Figure S6D, Supporting Information). These alterations of *cyclin* and *E2F* family gene expressions in HEK293‐TP53^KO^, HEK293‐TP53^KO^/NEK2 OE, H929‐TP53^KO^, and H929‐TP53^KO^/NEK2 OE cells were also verified by qPCR (Figure S6E, Supporting Information). It was previously reported that *NEK2* can be regulated by E2F1, E2F3, and E2F4 and that these genes are targets of p53.^[^ ^19^ ^]^ By using GEP data (GSE2658) mining, we found that the expression of *E2F8*, but not other *E2Fs*, positively and strongly correlates with *NEK2* expression in MM samples (*r* = 0.79, Figure 6D, Figure S6F, Supporting Information). *TP53* deletion in H929‐TP53^KO^ cells caused the mRNA and protein levels of *E2F8* to increase significantly when compared with the control cells (Figure 6E). In contrast, p53 overexpression in ARP1 cells (ARP1‐TP53 OE) decreased *E2F8* mRNA and protein levels (Figure 6F). It was previously shown that E2Fs can promote *NEK2* transcriptional activity by binding to the E2F site in the *NEK2* promoter region (−169 to −179 bp)^[^ ^19^ ^b]^ (Figure S1B, Supporting Information). We found that E2F8 overexpression in H929‐Ctrl and H929‐TP53^KO^ cells significantly upregulated *NEK2* levels (Figure 6G,H). Similarly, E2F8 overexpression in ARP1‐EV and ARP1‐TP53 OE cells also significantly upregulated *NEK2* expression, while *NEK2* levels were markedly lower in ARP1‐TP53 OE cells than in ARP1‐EV cells with E2F8 overexpression (Figure 6I,J). Additionally, ChIP‐qPCR analysis showed that the E2F8 binding site in the *NEK2* promoter was enriched in ARP1 cells (Figure 6K). Thus, p53 can also inhibit *NEK2* transcription via the regulation of cell cycle pathway‐related genes such as *E2F8* in MM (Figure 6L).

### 2.7 TP53 Suppresses *NEK2* Expression via Downregulation of DNA Methyltransferases

**Figure 7:**
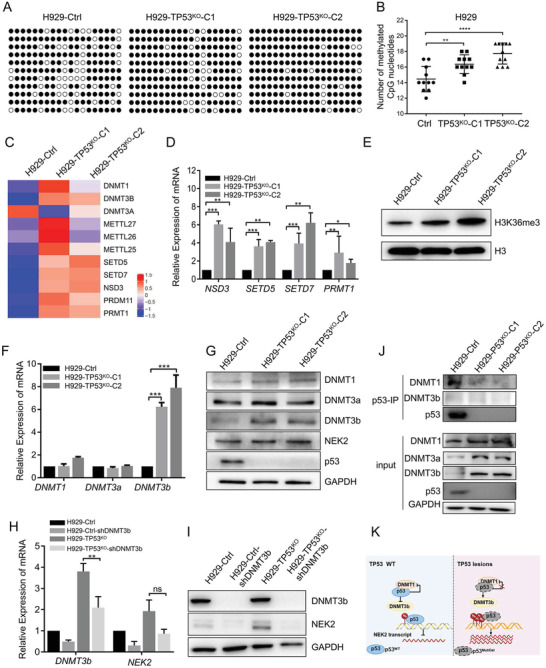
p53 suppresses *NEK2* expression by regulating the expression of DNMTs. A) Detailed BGS analysis confirmed methylation status of the *NEK2* promoter's distal CpG island in H929 cells with or without p53 deletion. B) Statistical analysis of methylated CpG dinucleotides in the *NEK2* promoter's distal CpG nucleotides in H929 cells with or without p53 deletion. Data presented as mean ± SD, *n* = 11, *p*‐values are calculated using one‐way ANOVA with Bonferroni correction, **p < 0.01, ****p < 0.0001. C) Heatmap of the ratios of the signal intensities of differential methylation‐related genes in H929 cells with or without p53 deletion. D) The mRNA levels of histone methylation‐related genes (*NSD3*, *PRMT1*, *SETD5*, and *SETD7*) detected by qPCR in H929 cells with or without p53 deletion. Data presented as mean ± SD, *p*‐values are calculated using unpaired t test, *n* = 3, *p < 0.05, **p < 0.01, ***p < 0.001. E) H3K36me3 protein levels detected using immunoblotting in H929 cells with or without p53 deletion. F,G) The mRNA and protein levels of *DNMT1*, *DNMT3a*, and *DNMT3b* in H929 cells with or without p53 deletion using qPCR and immunoblotting. Data presented as mean ± SD, *p*‐values are calculated using unpaired t test, *n* = 3, ***p < 0.001. H,I) The mRNA and protein levels of *NEK2* after shRNA‐mediated DNMT3b knockdown in wild type p53 and p53‐deleted H929 cells, detected by qPCR and immunoblotting. Data presented as mean ± SD, *p*‐values are calculated using unpaired t test, *n* = 3, **p < 0.01. J) Endogenous p53 was pulled down by p53 antibodies in H929 cells with or without p53 deletion, and the DNMT1, DNMT3a, and DNMT3b proteins were analyzed by western blotting. The lysates before IP were used as a positive control. K) Dysfunctional p53 elevates *NEK2* expression in MM by upregulating DNMT expression, thereby increasing methylation of the *NEK2* promoter.

It was previously reported that p53 represses *NEK2* expression and protects its binding region from accumulating DNA methylation.^[^ ^18^ ^]^ We also found that DNA methylation was elevated in H929 cells with *TP53* deletion (TP53^KO^‐C1 and TP53^KO^‐C2; Figure S7A,B, Supporting Information). To explore how p53 represses *NEK2* expression through DNA methylation, we first examined the levels of DNA methylation‐related genes (*DNMT1*, *DNMT3a*, *DNMT3b*) and histone methylation‐related genes (*NSD3*, *PRMT1*, *SETD5*, and *SETD7*) and found that they were upregulated in H929‐TP53^KO^ compared with H929‐Ctrl cells (**Figure** **7**C,D). Upregulation of the histone methylation‐related genes (*NSD3*, *PRMT1*, *SETD5*, and *SETD7*) caused H3K36me3 protein methylation (Figure 7E). Subsequently, we showed that the expression of *DNMT3b* was significantly upregulated compared with the expression of the other two DNA methyltransferase genes (*DNMT1* and *DNMT3a*) in H929‐TP53^KO^ cells (Figure 7F,G). Aberrantly high expression of *DNMT3b* and *DNMT1* coincided with upregulated *NEK2* expression (Figure S7B, Supporting Information). When *DNMT3b* and *DNMT1* were depleted via shRNA, *NEK2* mRNA and protein expression levels were dramatically downregulated in H929 cells with or without p53 (Figure 7H,I, Figure S7C–F, Supporting Information), while lower *NEK2* expression levels were found in H929‐Ctrl cells expressing wild type p53. Additionally, a co‐immunoprecipitation (co‐IP) assay showed that p53 interacted with DNMT1 and DNMT3a, but not with DNMT3b, in H929 cell lines (Figure 7J). We also confirmed that *NEK2* methylation levels decreased in p53‐overexpressing ARP1 cells (Figure S7F, Supporting Information). These results suggested that p53 protects its binding region in the *NEK2* promoter from accumulating DNA methylation by repressing DNMT expression (Figure 7K).

### 2.8 TP53 Overexpression Synergistically Interacts with NEK2 Suppression to Promote Tumor Formation and Reduce Bortezomib Sensitivity

**Figure 8:**
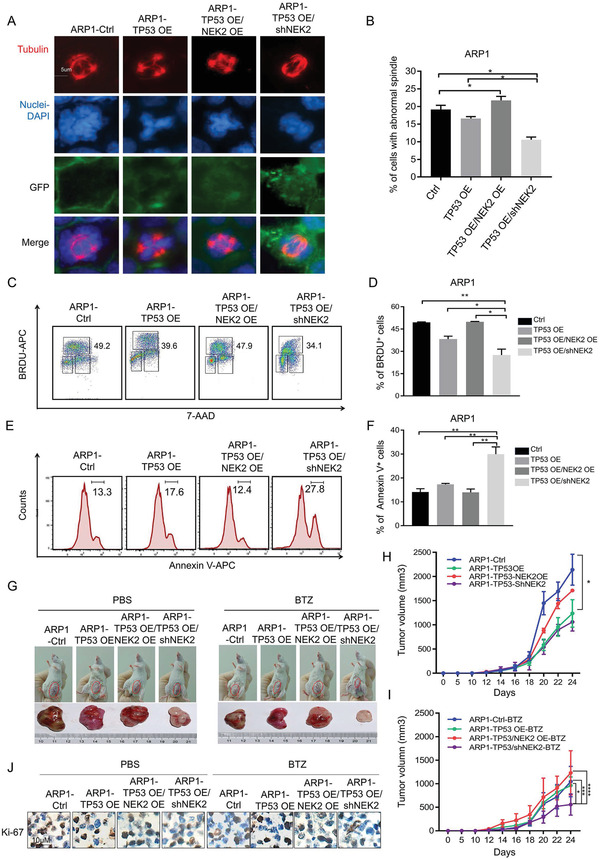
Downregulation of NEK2 in *TP53*‐deleted MM cells inhibits cell growth and decreases drug resistance. A,B) Representative images of spindles (Red, Tubulin), GFP (Green), and nuclei (Blue) in ARP1‐Ctrl, ARP1‐TP53 OE, and ARP1‐TP53 OE/shNEK2 cells. Quantifications show the percentage of abnormal monopolarity from three independent experiments. At least 60 spindles were randomly chosen and counted in each experiment. All results are shown as means ± SD of three independent experiments, Data presented as mean ± SD, p‐values are calculated using one‐way ANOVA with Dunnett post‐hoc test, *p < 0.05. Flow cytometric detection of BrdU‐positive cells in ARP1‐Ctrl, ARP1‐TP53 OE, and ARP1‐TP53 OE/shNEK2 cells. Representative C) dot plots and D) quantification. Data presented as mean ± SD, *p*‐values are calculated using one‐way ANOVA with Dunnett post‐hoc test, *n* = 3, *p < 0.05, **p < 0.01. E) Representative histograms for detection of apoptotic cells and F) statistical analysis of the percentage of apoptotic cells in ARP1‐Ctrl, ARP1‐TP53 OE, ARP1‐TRP53 OE/NEK2 OE, and ARP1‐TP53 OE/shNEK2 cells treated with 4 nM BTZ for 48 h. Data presented as mean ± SD, *p*‐values are calculated using one‐way ANOVA with Dunnett post‐hoc test, n = 3, **p < 0.01. G) Representative images of tumor xenografts in B‐NDG mice after subcutaneous injection of ARP1‐Ctrl, ARP1‐TP53 OE, ARP1‐TP53 OE/NEK2 OE, or ARP1‐TP53 OE/shNEK2 cells into the right abdomen. Cells were treated with BTZ or with PBS as a negative control. Tumor volumes of xenografts derived from B‐NDG mice injected with ARP1‐Ctrl, ARP1‐TP53 OE, ARP1‐TP53 OE/NEK2 OE, or ARP1‐TP53OE/shNEK2 cells (*n* = 4). Cells were treated I) with BTZ or H) with control. Data presented as mean ± SD, *p*‐values are calculated using two‐way ANOVA with Dunnett post‐hoc test, *n* = 6, *p < 0.05, **p < 0.01, ***p < 0.001. J) Representative images for IHC detection of Ki‐67 protein in the tumor nodules derived from B‐NDG mice injected subcutaneously with ARP1‐Ctrl, ARP1‐TP53 OE, ARP1‐TP53 OE/NEK2 OE, or ARP1‐TP53 OE/shNEK2 cells. Cells were treated with BTZ (right) or with control (left).

We next determined the effect of combined p53 overexpression and NEK2 suppression on chromosome stability, proliferation, and drug resistance in TP53^−/−^ MM cell lines using in vitro and in vivo assays. Spindle formation assay experiments showed that the number of cells with abnormal mitosis were reduced after p53 ectopic expression in ARP1 cells (ARP1‐TP53 OE, 17.6 ± 0.7%) compared with control cells (ARP1‐EV, 19.2 ± 0.9%). Meanwhile, the number of cells with mitotic abnormalities was elevated after NEK2 overexpression in ARP1‐TP53 OE cells (ARP1‐TP53 OE/NEK2 OE, 21.7 ± 0.8%). However, the number of defective cells was substantially reduced when NEK2 was depleted by shRNA‐mediated knockdown in ARP1‐TP53 OE cells (ARP1‐TP53 OE/shNEK2, 10.3 ± 0.7%) (**Figure** **8**A,B). Similarly, BrdU intake assay experiments revealed that the number of BrdU‐positive cells decreased significantly in the ARP1‐TP53 OE group, but increased in the ARP1‐TP53 OE/NEK2 OE group, compared with ARP1‐EV cells. In contrast, the number of defective cells decreased dramatically in ARP1‐TP53 OE/shNEK2 cells compared to ARP1‐EV or ARP1‐TP53 OE cells (Figure 8C,D).

Subsequently, we tested the sensitivity of cells with combined p53 and NEK2 depletion to BTZ by using an apoptosis assay. We found that the percentage of apoptotic cells increased significantly in the ARP1‐TP53 OE/shNEK2 group (30.0 ± 3.04%) after 4 nM BTZ treatment when compared with the ARP1‐Ctrl (14.1 ± 1.41%) and ARP1‐TP53 OE (17.3 ± 0.42%) groups (Figure 8E,F).

In addition, by using an MM xenograft model with B‐NDG immunodeficient mice, we confirmed that the tumor sizes produced from ARP1‐TP53 OE cells were much smaller than those from ARP1‐EV or ARP1‐TP53 OE/NEK2 OE cells (Figure 8G,H). Furthermore, tumors formed by ARP1‐TP53 OE/shNEK2 cells were the smallest and were much more sensitive to BTZ than were ARP1‐TP53 OE cells (Figure 8G,I). Subsequent IHC analysis also revealed that cells in tumor nodules were CD138^+^ (Figure S8A, Supporting Information), and the number of Ki‐67^+^ cells was reduced, suggesting that the recovery of p53 function and further suppression of NEK2 are beneficial for the p53‐deleted group of cells (Figure 8J, Figure S8B, Supporting Information). Thus, stable expression of wild type p53 rescues the defects in mitosis, proliferation, and tumorigenesis in MM cells and enhances the therapeutic effect of BTZ both in vitro and in vivo. This rescue effect can be further improved when combined with NEK2 depletion.

## 3 Discussion

Collaboration between oncogenes and tumor suppressor genes is an important mechanism in the development of MM. Here, we showed that *NEK2* amplification is a major cause for *NEK2* upregulation in MM and other cancer types, especially in *TP53^−/^* ^−^ MM patients. Previously, we had reported that NEK2 overexpression induces drug resistance, proliferation and CIN in cancer cells.^[^ ^11^ ^]^ In this study, we found that patients with combined defects of *NEK2* activation and p53 inactivation suffer from poor survival and that collaboration between *NEK2* and *TP53* defects augments MM cell growth and drug resistance in vitro and in vivo.

Gene mutations, DNA amplification and promoter methylation status are the major causes for aberrantly high expression of candidate tumor genes.^[^ ^17^ ^a,^ ^17^ ^]^ Previous studies focused on the function, mechanisms and protein stability of NEK2, but how *NEK2* is activated and upregulated remained poorly understood. In this study, using a FISH probe for *NEK2* DNA, we examined the copy number of *NEK2* in MM cells and patients and assessed mutations in the *NEK2* promoter and exons. We further examined DNA methylation in the proximal and distal *NEK2* promoter regions in MM cell lines and analyzed the effect of *NEK2* mutation on its expression (data not shown) using genomic data from a recent MMRF study (study accession phs000748). Interestingly, we found that *NEK2* is only amplified in the MM cells of patients who have a poor rate of survival, suggesting that *NEK2* amplification is the major cause for *NEK2* activation in MM. To date, *NEK2* DNA amplification in tumors is still poorly documented due to a lack of appropriate FISH probes for detecting the chromosomal copy number of *NEK2*. In our present study, we prepared a DNA probe of *NEK2* to detect its copy number with FISH, leading us to find that *NEK2* amplification occurs in 23.5% of MM (AD) patients, similar to the data from MMRF study. Thus, it is highly likely that our prepared DNA probe can be used for clinical detection of *NEK2* amplification in the future.

The 17p chromosomal region harbors the gene locus of *TP53*, an important tumor suppressor gene.^[^ ^21^ ^]^ Deletion of this region is a recurrent cytogenetic abnormality present in 10–34% of MM cases along with disease progression and is considered an independent factor responsible for less favorable clinical outcome in MM patients.^[^ ^2^ ^a,^ ^4^ ^b]^ In keeping with this notion, the lesions associated with short OS in multivariate analysis are +1q and del17p13 in MM,^[^ ^21^, ^22^ ^]^ suggesting that the combined cytogenetic abnormality contributes to the progression of MM. Despite these advances, the molecular mechanisms underlying p53's action are poorly understood. MDM4, a homolog of MDM2, is located in the +1q region and inactivates p53 by binding to and inhibiting its transactivation.^[^ ^22^ ^a,^ ^23^ ^]^ Whether this occurs in MM is unknown. Our current studies demonstrated that *NEK2* amplification correlates strongly with *TP53* deletion in MM and has a significant clinical impact. Previously, it had been reported that NEK2 and p53 modulate each other. On the one hand, methylation of the distal *NEK2* promoter containing the p53‐binding site affects p53 binding to the promoter, resulting in the inability of p53 to attenuate *NEK2* expression.^[^ ^18^ ^]^ On the other hand, NEK2 can attenuate the function of wild type p53 by inhibiting its phosphorylation.^[^ ^23^ ^]^

**Figure 9:**
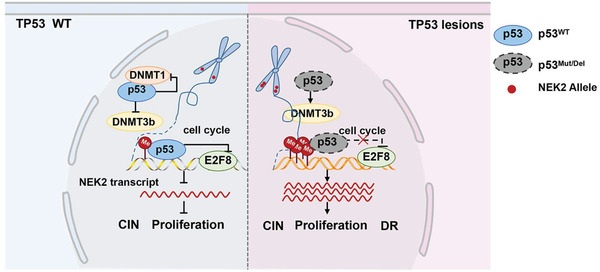
Working model depicting the regulation of *NEK2* expression by *TP53*.

In this study, we found that, under normal physiological conditions, p53 is an active protein that binds to the transactivation domain in the *NEK2* promoter. This inhibits *NEK2* transcription through the cell cycle pathway mediated by p53‐p21‐DREAM‐E2F and protects its distal CpG island from increased methylation. The E2F transcriptional factors mediate various biological functions involved in cell cycle progression.^[^ ^24^ ^]^ Previous studies suggested that cell cycle arrest is achieved through indirect transcriptional repression by p53‐p21‐DREAM‐E2F/CHR.^[^ ^25^ ^]^ In this pathway, the target genes with E2F or CHR promoter sites are transcriptionally regulated by the DREAM transcriptional inhibitory complex.^[^ ^25^ ^]^ One E2F binding site in the *NEK2* promoter region (−169 to −179 bp) was previously reported.^[^ ^19^ ^b]^ Our studies demonstrated, for the first time, that E2F8 serves as a transcription factor to induce *NEK2* expression through the p53/E2F8 pathway in MM. It is well‐known that chromatin opening and gene transcriptional activation are regulated by the status of histone modifications. H3K36 methylation, which is regulated by histone methylation modification‐related genes such as *NSD3*, *PRMT1*, *SETD5*, and *SETD7*, can promote gene transcriptional activation.^[^ ^26^ ^]^ Our results showed that mRNA levels of these genes and H3K36me3 protein levels increase in *TP53*‐knockout MM cells (H929‐TP53^KO^). In mammals, DNA methylation is mainly catalyzed by DNMTs including the DNMT1, DNMT2 and DNMT3 families. DNMT1 is a maintenance methylase, while DNMT2 can bind to specific heterotopic sites on DNA, but its specific role is not clear. DNMT3a and DNMT3b are re‐methylases that re‐methylate demethylated CpG sites to participate in *de novo* methylation of DNA. In this study, we found that the methylation of CpG sites within the *NEK2* promoter distal CpG island, which are the p53 binding sites reported in previous studies,^[^ ^18^ ^]^ was elevated in *p53*‐deleted MM cells. The mRNA and protein levels of *DNMT3b* and DNMT1 protein were also upregulated significantly. In addition, co‐IP experiments showed that p53 interacts with DNMT1. The expression of *DNMT1* and *DNMT3b* was highly positively correlated to increased *NEK2* expression, and the mRNA and protein levels of *NEK2* were decreased when DNMT1 or DNMT3b was knocked down with shRNA, especially in DNMT3b depleted MM cells. In this study, p53 inhibited the expression of the *DNMT3b* and DNMT1 proteins, reducing methylation of the *NEK2* promoter CpG island. Thus, wild type p53 binds to *NEK2* DNA and inhibits its transcription. These results suggest that p53 suppresses *NEK2* expression by regulating DNMT expression, thereby protecting its binding region from accumulating DNA methylation. Together, when *TP53* has gain‐of‐function mutation or loss, the dysfunctional p53 elevates *NEK2* expression genetically by inducing *NEK2* amplification, transcriptionally by mediating E2F8 activity and epigenetically by repressing DNMT expression (**Figure** **9**).

We found that p53 regulates *NEK2* at both the genetic and the transcriptional levels in MM based on the following observations. First, at the clinical level, deletion of the *TP53*‐containing chromosome occurs in 9.3% (51/548) of MM samples and correlates with aberrantly high expression of *NEK2*. Indeed, we found *NEK2* amplification in 25.5% (13/51) of MM samples, and it was closely linked to high‐level expression of *NEK2*. Furthermore, experiments with TCGA data mining showed that *TP53* deletion correlates strongly with *NEK2* amplification and aberrantly high expression in several cancer types. Thus, *TP53* correlates inversely with *NEK2* amplification and overexpression in patients with MM and other cancer types. Second, at the genetic level, we used CGH and FISH analyses to observe *NEK2* amplification in *TP53^−/^* ^−^ cell lines. Third, at the transcriptional level, experiments with the luciferase reporter assay and ChIP‐qPCR confirmed that p53 binds directly to the *NEK2* promoter in MM cells. Wild type p53 inhibits *NEK2* transcription, while mutant p53 significantly increases its expression. Finally, at the functional level, combined *TP53* deletion and *NEK2* overexpression in WT *TP53* MM cell lines induce asymmetric mitosis and promote proliferation and tumorigenic activity in vitro and in vivo. Conversely, concomitant p53 overexpression and NEK2 inhibition in *TP53^−/−^* cells can partially rescue these defects, including drug resistance, in MM cells in vitro and in vivo.

A preclinical study using myeloma demonstrated that adenovirus‐mediated delivery of WT *TP53* can potently induce apoptosis.^[^ ^27^ ^]^ Our current study also suggests that delivery of WT *TP53* and inhibition of *NEK2* in a *TP53* ^−/−^ MM cell line can suppress tumor formation and enhance BTZ's therapeutic effect. Our findings suggest that targeting the function of the NEK2 and p53 pathways may have therapeutic values by reversing the adverse outcome of MM patients without p53.

In conclusion, we found that *NEK2* amplification leads to *NEK2* upregulation, while combined defects in *TP53* and *NEK2* can be used as a novel marker for poor prognosis in a cohort of MM patients. We also validated NEK2 as a novel therapeutic target in the *TP53^−/−^* subset of MM and revealed a novel mechanism by which *TP53* regulates *NEK2* at both the genetic and transcriptional levels. Future experimentation should examine the clonal evolution of the subgroup containing *TP53* and *NEK2* dual defects and their functions in MM initiation, promotion and drug resistance. Selective inhibitors should be developed to improve the clinical outcomes for these patients.

## 4 Experimental Section

### Human Samples

Human bone marrow samples were obtained from healthy donors (*n* = 8) and newly diagnosed (*n* = 82) and relapsed (*n* = 25) MM patients in the Third Xiangya Hospital, Central South University (Changsha, China) and Institute of Hematology and Blood Disease Hospital, Chinese Academy of Medical Sciences and Peking Union Medical College (Tianjin, China). Written informed consent was obtained from all participants. The sampling procedure was approved by the Cancer Research Institute of the Central South University Medical Ethics Committee. Primary MM cells and normal plasma cells were isolated from the mononuclear cells of BM samples using CD138 MicroBeads (Miltenyi Biotec, Auburn, CA).

### Cell Culture and Reagents

The human MM cell lines H929, MM.1S, MM.1R, RPMI‐8226, and U266 were purchased from the American Type Culture Collection (ATCC, Manassas, VA). HEK293, ARP1, and OCI‐my5 cell lines were obtained from the Cancer Research Institute of Central South University. HCT116‐TP53^WT^ and HCT116‐TP53^−/−^ cells were provided by Dr. Tiebang Kang (Sun Yat‐sen University Cancer Center, Guangzhou, China) and maintained in McCoy's 5A medium (#16 600 082, Gibco‐BRL, Grand Island, NY, USA) supplemented with 10% fetal bovine serum (FBS) (#04‐001‐1, Biological Industries (BI), Kibbutz Beit‐Haemek, Israel) and 1x penicillin and streptomycin (P/S) (#15140‐122, Gibco‐BRL). All MM cell lines were maintained in RPMI‐1640 medium (#C11875500BT, Gibco‐BRL, Suzhou, China) supplemented with 10% FBS and 1x P/S. HEK293 cells were identified by STR analysis (Supporting information 2) and maintained in DMEM medium (#01‐051‐1ACS, BI) supplemented with 10% FBS and 1x P/S.

Anti‐NEK2 (D‐8, #sc‐5560), anti‐p21 (F‐5, #sc‐6246), and anti‐E2F2 (KH95, #sc‐251) and mouse or rabbit secondary antibodies were from Santa Cruz Biotechnology (CA, USA). Anti‐p53 (#10442‐1‐AP), anti‐E2F8 (#13425‐1‐AP), and anti‐GAPDH (#10494‐1‐AP) antibodies were purchased from Proteintech (Wuhan, Hubei, China), while nutlin‐3a and doxycycline were purchased from Selleckchem (#S8059, Houston, TX, USA). Doxorubicin was from Harbin Pharmaceutical Group Bioengineering Co. (Harbin, China).

### Vectors and Transfections

*NEK2*, *E2F8*, and wild type and mutant *TP53* cDNAs containing open reading frames (ORF) were subcloned into the pCDH vector tagged with GFP or RFP (#CD511B‐1, Addgene, Watertown, MA, USA). *NEK2*, *E2F8*, and wild type *TP53* ORF sequences (Table S10, Supporting Information) were amplified by RT‐PCR using RNA purified from HEK293 cells as a template with TP53‐ORF primers and confirmed by sequencing. Mutant *TP53* expression vector was obtained from wild type *TP53* expression vector by site‐directed mutagenesis (Supporting Information 3). The shRNAs (shRNA sequence for *NEK2* and *E2F8* is shown in Table S10, Supporting Information) were inserted into EcoRI and Xhol sites of the pLKO vector tagged with Tet and puromycin (Addgene). Lentiviruses were packaged in HEK293T cells using pMD2G and psPAX2 helper vectors and polybrene (3 µg mL^−1^) ‐mediated transduction (#H9268‐5G, Sigma‐Aldrich, Missouri, USA). Transient transfection was performed using Lipofectamine 3000 reagent (#L3000015, Invitrogen, California, USA) according to the manufacturer's instructions. *NEK2* and *TP53* overexpressing cells were sorted with flow cytometry by GFP or RFP 5 days post‐infection. Cells expressing *NEK2* shRNAs were selected with 1–2 µg mL^−1^ puromycin (#A1113803, Invitrogen) for 24 h.

*TP53* homozygous gene knockout cell lines were established with the CRISPR‐Cas9 system. CRISPR‐Cas9‐Lenti‐V2‐TP53, including four sgRNA guide sequences and CRISPR‐Cas9‐Lenti‐V2 (as a control), was provided by Dr. Tiebang Kang (Sun Yat‐sen University Cancer Center). 3 × 10^5^ HEK293 cells were transfected with four different sgRNA guide sequences, and cells were cultured for 3 days. After western blot analysis showed the p53 protein level in transfected cells was shown to significantly decrease, the target sgRNA was selected to subsequently establish cell lines. Transfected HEK293 cells were selected with puromycin (1 µg mL^−1^) for 24 h and then maintained continuously with 0.5 µg mL^−1^ puromycin. Small single colonies emerging from single cells were picked and expanded to derive isogenic cell lines with defined mutations. To obtain H929 cell lines with *TP53*‐knockout by using CRISPR, *TP53* sgRNA was inserted into the pL‐CRISPR.EFS.GFP vector provided by Jiaxi Zhou (State Key Laboratory of Experimental Hematology). Next, H929 cells were infected with lentiviruses containing pL‐CRISPR.EFS.GFP ‐TP53 plasmids and sorted by GFP 5 days after infection. Single colonies were obtained by sequential dilution after sorting (Supporting Information 3). Deletion of the *TP53* gene in selected HEK293 and H929 cell clones was confirmed by qPCR and western blotting. The sgRNA guide sequences and genotyping primers are listed in Table S10, Supporting Information.

### Fluorescence In‐Situ Hybridization

FISH was performed on interphase nuclei using established methods.^[^ ^11^ ^]^ As previously described, all MM cell samples were purified using Miltenyi technology (anti‐CD138‐coated magnetic beads) before FISH. To detect *NEK2* amplification, a *CEP1* probe targeting chromosome 1 (#CHR01‐10‐GR, Empire Genomics, Buffalo, NY, USA) and a bacterial artificial chromosome (BAC) at 1q32.2 (#RP11‐1114G13, Invitrogen) were purchased. The BAC probe was labeled using the Nick Translation Kit (#32‐801300, Abbott, Chicago, USA) with green‐dUTP (#02N32‐050, Abbott) or orange PF555‐dUTP (#PK‐PF555‐8‐100, PromoKine, Germany). The status of the *TP53* gene was analyzed using a DNA probe targeting *TP53* (#TP53‐20‐OR, Empire Genomics). Interphase FISH was performed according to the procedure described previously. Briefly, the probes were hybridized to CD138^+^ cells. The slides were stored at 4 °C until FISH analyses were performed. Interphase FISH signals were evaluated in at least 200 interphase nuclei in each sample. If at least three copies were seen in at least 20% of CD138^+^ cells, it was considered evidence of gain/amplification. To investigate effects of the magnitude of AmpNEK2 on clinical outcomes and each category, AmpNEK2 was divided into two categories: 1) Three copies of the *NEK2* probe (the percentage of clonal plasma cells with at least three copies was <20%) and 2) more than three copies of *NEK2* probe (the percentage of clonal plasma cells with more than three copies was ≥ 20%).

### Soft Agar Clonogenicity Assay

1000 cells per well were seeded in 12‐well plates for double‐layer agar cultures for 3 weeks. Cells were resuspended in 0.3% agar (#16 520 100, Invitrogen) in RPMI‐1640 medium supplemented with 15% FBS. Cells were incubated (37 °C, 5% CO_2_) and fed with the same medium every three days on the up‐layer for three weeks. The aggregates of cells > = 50 cells were defined as colonies. Photographs of all plates were scanned with ChemiDoc XRS (Bio‐Rad, California, USA), and the colonies were counted using ImageJ (NIH, USA).

### BrdU Assay

For the BrdU assay, all procedures followed the standard protocol with the APC BrdU Flow Kit (#552 598, BD, New Jersey, USA). Cells were labeled with BrdU in culture medium for 1 h. Subsequently, the incorporated BrdU was stained with specific anti‐BrdU fluorescent antibodies, and 7‐aminoactinomycin D (7‐AAD) was used to label total DNA in conjunction with BrdU staining. Finally, cells stained with BrdU and 7‐AAD were examined by flow cytometry and analyzed with FlowJo 10.0 software.

### Apoptosis

Apoptotic cells were labeled by APC‐conjugated Annexin V (BD). Dead cells were labeled by 7‐AAD (BD). Cell staining was performed according to the manufacturer's protocol. Labeled cells were then measured by CytoFLEX (Beckman Instruments, Inc, CA, USA). The percentages of apoptotic cells were calculated using FlowJo software.

### Immunofluorescence

Bone marrow aspirates from human myeloma patients were sorted with anti‐CD138 magnetic beads and mounted onto cytospin slides for this study. Myeloma cells were fixed in 4% formaldehyde, and primary antibodies against NEK2 (mouse anti‐human, D‐8, #sc‐55601, Santa Cruz Biotechnology) and p53 (rabbit anti‐human, #10442‐1‐AP, Proteintech) were added at a final dilution of 1:100 followed by overnight incubation at 4 °C. The secondary antibodies goat anti‐rabbit Alexa Fluor 488 (#A21202, Invitrogen) and goat anti‐mouse Alexa Fluor 594 (#A21207, Invitrogen) were added at a final dilution of 1:1000 for 1 h at room temperature. The slides were washed and mounted with DAPI. Images were captured using a confocal microscope (Nikon, Tokyo, Japan).

### Luciferase Activity Assay

The *NEK2* promoter sequence ranging from −1017bp to −2 bp, with or without the p53 binding site, was inserted into a pGL3‐enhancer vector (Promega, Wisconsin, USA) and subsequently a luciferase reporter gene vector. The luciferase reporter gene constructs were named pGL3‐NEK2‐P6 and pGL3‐NEK2‐P6M, respectively. Subsequently, pGL3‐control (positive control, Promega), pGL3‐NEK2‐P6, pGL3‐NEK2‐P6M, and the internal control Renilla (pRL‐null, #E2271, Promega) vectors were co‐transfected with wild type pcDNA3.1‐TP53‐FLAG and the *TP53* expression vector into HEK293‐TP53^KO^ and ARP1 cells using Lipofectamine 3000. Cells were harvested after 48 h cultivation, and a luciferase activity was detected using Dual‐Luciferase Reporter Assay System (Promega) and a GloMax 20/20 Luminometer (Promega) according to the manufacturer's protocol. All samples were done in triplicate. The primers for luciferase reporter gene constructs are listed in Table S10, Supporting Information.

### Chromatin Immunoprecipitation

The binding of the *NEK2* subunit to DNA in *TP53*‐knockout H929‐T53^KO^ and wild type *TP53* H929‐Ctrl MM cell lines was quantified with ChIP‐quantitative PCR (ChIP‐qPCR). The chromatin immunoprecipitation (ChIP) assay was performed with the EZ‐ChIP kit (#17‐371 RF, Millipore, Massachusetts, USA), Briefly, chromatin (5 µg) from the two myeloma cell lines was used in the ChIP assay using antibodies (3 µg) against p53 (DO‐1, #sc‐126 X, Santa Cruz Biotechnology) or E2F8 (#13425‐1‐AP, Proteintech). The ChIP DNA fragments were quantified with the EZ‐ChIP kit and the enrichment of DNA fragments containing putative p53 binding sites in the gene promoter was quantified by qPCR using a LightCycler 96 (Roche, Basel, Switzerland). Two specific primers for *NEK2*, *p21*, and *GAPDH* (Table S10, Supporting Information) were used to amplify fragments containing the predicted p53 or E2F8 binding sites in the *NEK2* promoter region. As a negative control, *GAPDH* was also amplified with the corresponding primers, while *p21* was used as a positive control. Values obtained from immunoprecipitated samples were normalized to that of their corresponding input samples. Data are representative of three separate experiments, and error bars indicate mean ± SD. The primers used in the ChIP‐qPCR assay are listed in Table S10, Supporting Information.

### Comparative Genomic Hybridization

A CGH array of HEK293‐TP53KO and HEK293‐NEK2 OE versus HEK293‐Ctrl cells was performed to identify, in combination with bioinformatics, amplified or deleted genes among these three cell types. The SurePrint G3 Human CGH Microarray Kit, 2 × 400K chip (Agilent, California, USA) to detect genome‐wide differences was used. After the completion of hybridization, the array slides were taken out and washed, then placed into an Agilent Microarray Scanner for scanning. After scanning, the data were interpreted with Agilent Feature Extraction software. The CGH differential region was then calculated using Agilent CytoGenomics software. The detection method is numbered AG‐GC‐WL01‐01‐2012, and the data analysis method is numbered AG‐GC‐DL01‐01‐2010. The whole testing process was completed by Capital Bio Technology (Shanghai, China).

### Assessment of Mitotic Spindle Phenotypes

Assessment of mitotic spindle phenotypes was performed according to the procedure described previously.^[^ ^28^ ^]^ Briefly, cells were fixed in 4% paraformaldehyde (#P1110, Solarbio, Beijing, China), permeabilized by 0.1% Triton X‐100 (Sigma‐Aldrich) and incubated with fluorescent anti‐*α*‐tubulin (#ab7291, Abcam, Cambridgeshire, UK) at 1:1000 dilution for 2 h, with the secondary antibodies goat anti‐mouse Alexa Fluor 594 (#A21207, Invitrogen) at 1:1000 dilution for 1 h and 0.1 µg mL^−1^ DAPI (#D9564‐10MG, Sigma‐Aldrich) for 5 min at room temperature. Cells were then analyzed by Axio Imager Z2+Metafer fluorescence microscopy (Zeiss, Oberkochen, Germany). Cells showing asymmetric spindle division, multipolar division, and nuclear condensation were counted in at least 60 cells in each sample and expressed as mean ± SD%.

### RNA Sequencing and Data Analysis

Total RNA was extracted from fresh cells using Trizol (#15 596 018, Invitrogen) and quality was assessed via NanoDrop (Thermo Fisher, New York, USA). Samples with total RNA greater than 300 ng and RIN (RNA integrity number) > 7 were retained for RNA‐seq. MM cell lines including HEK293‐Ctrl, HEK293‐TP53^KO^, and HEK293‐TP53^KO^/NEK2 OE were used for RNA‐seq. The total RNA sample was used to construct complementary DNA libraries according to the TruSeq RNA Library Prep Kit (Illumina, CA, USA) protocol. RNA‐seq was performed on a HiSeq 2000 (Illumina) and pair‐end sequencing data was generated. Sequence files for all samples used in this study have been deposited in the public database of National Omics Data Encyclopedia (NODE) under project number OEP000456, available at: https://www.biosino.org/node/review/detail/OEV000066?code=UZQODSSG.

All sequencing reads were aligned with the reference genome (GRch38) using HISAT2,^[^ ^29^ ^]^ with default options in the StringTie^[^ ^30^ ^]^ RNA‐seq workflow.^[^ ^31^ ^]^ After the removal of improperly aligned reads, read count information was extracted from the files generated by StringTie with a provided Python script (prepDE.py). Finally, EdgeR^[^ ^32^ ^]^ in R was used to screen differentially expressed genes in the samples.

### Mouse Xenograft Models

All animal work was performed in accordance with the guidelines of the Institutional Animal Care and local veterinary office and ethics committee of the CSU, China (Animal experimental license, NO.2019sydw0146) under approved protocol.

For Figures 4 and 8, 1 × 10^6^ H929‐Ctrl, H929‐NEK2 OE, H929‐TP53^KO^, H929‐TP53^KO^/NEK2 OE, or H929‐TP53^KO^/shNEK2 cells (in 150 µL PBS) were injected subcutaneously into the abdomen of immunodeficient female B‐NDG (6–8 weeks old) mice without mature T cells, B cells, and NK cells^[^ ^33^ ^]^ (Biocytogen Co, Beijing, China). 1 × 10^6^ ARP1‐Ctrl, ARP1‐TP53 OE, ARP1‐TP53/NEK2 OE, or ARP1‐TP53 OE/shNEK2 cells (in 150 µL PBS) were injected subcutaneously into the abdomen of 6–8 weeks old B‐NDG mice (Biocytogen Co, Beijing, China). After 14 days, mice with ≈3 × 3 mm tumor nodules were treated with 1 mg kg^−1^ BTZ every three days until the end of experiment. Mice injected with ARP1‐TP53 OE/shNEK2 cells were fed with water containing 2 mg mL^−1^ doxycycline every 2 days when the size of tumor nodule was about 3 × 3mm.

Tumor burdens were monitored by measuring tumor volumes every 3 days. Tumor volumes were calculated according to the equation *V* = (length × width^2^)/2. Expression of NEK2, p53, and Ki‐67 proteins in tumor nodules were detected via immunohistochemistry.

### Immunohistochemistry Staining

Tumor xenografts derived from mice in vivo were fixed in formalin for 48 h. Then, microarray slides were cut at four microns on plus slides. IHC was performed using a standard streptavidin‐biotin‐peroxidase complex method as previously described. Slides were allowed to air dry and placed in a 55–60 °C oven for 30 min. The tissue sections were incubated with the primary antibodies anti‐CD138 (#10593‐1‐AP, Proteintech), anti‐NEK2 (D‐8, #sc‐55601, Santa Cruz Biotechnology), p53 (#AMO183, Spectre, Xiamen, China), and Ki‐67 (#AMO383, Spectre) at a final dilution of 1:100 overnight at 4 °C. Assessment of the stained proteins was carried out by determining both the intensity (0, 1, 2, or 3) and extent of staining (0, 0%; 1, <10%; 2, 10–50%; 3, >50%) as previously described.^[^ ^34^ ^]^

### Accession Numbers

Microarray data sets were deposited in the National Center for Biotechnology Information's Gene Expression Omnibus (GEO) database (http://www.ncbi.nlm.nih.gov/geo) using the accession numbers GSE2658^[^ ^5^ ^]^ .The MMRF CoMMpass database were based on the Relating Clinical Outcomes in Multiple Myeloma to Personal Assessment of Genetic Profile study (CoMMpassSM, NCT01454297). The MMRF genomic data can be found on the GDC Data Portal. To request access to protected MMRF data, please apply to dbGaP for access to the MMRF Study (study accession phs000748).

### Real‐Time Quantitative PCR

For quantitative analysis of gene expression, total RNA was isolated with Trizol. Complementary DNA was synthesized using a first full cDNA transcription kit according to the manufacturer's instructions (#K1622, Thermo Fisher). Real‐time qPCR for human *NEK2*, *TP53*, *E2F8*, *GAPDH*, and other genes listed below was performed using SYBR Green Super Mixture Reagents (#A25742, Invitrogen) on a LightCycler 96 system (Roche). PCR was initiated at 95 °C for 2 min to hot‐start the DNA polymerase and denature the template, followed by 40 cycles of denaturing at 95 °C for 15 s and annealing and extension at 60 °C for 1 min. The primers used in the qPCR assay are listed in Table S10, Supporting Information.

### Immunoblotting

Cell pellets were lysed with RIPA lysis buffer and 20–40 µg protein from each experimental condition were subjected to sodium dodecyl sulfate‐polyacrylamide gel electrophoresis, which was then transferred onto a polyvinylidene fluoride membrane. The membranes were blocked with 5% nonfat dry milk in Tris‐buffered saline solution containing 0.1% Tween 20 (TBS‐T) for 1 h at room temperature and then incubated with the appropriate primary antibodies overnight at 4 °C. Primary antibodies were diluted in 5% bovine serum albumin with TBS‐T. The membranes were washed with TBS‐T, followed by probing with species‐specific secondary HRP‐conjugated antibodies conjugated, which were diluted in bovine serum albumin with TBS‐T. Protein bands were detected using SuperSignal West Pico Chemiluminescent Substrate (Pierce, Rockford, IL, USA)

### Methylation‐Specific PCR

MSP was performed using our previously published protocol.^[^ ^35^ ^]^ Briefly, MSP was carried out for 45 cycles using the Ex Taq Hot Start DNA polymerase (#RR006A, TaKaRa, Dalian, China) with 10 ng of sodium bisulfite‐treated DNA. MSP products (both U and M products) of NEK2 from partial samples, which accounted for 20% of all detected samples, were purified with Agarose Gel Purified System (#LS1022, QIAGEN, Promega) and subsequently sequenced. All sequencing reactions were performed by a 377 ABI PRISM DNA Sequencer at the Shanghai Invitrogen Company (Shanghai, China). The primers used in the MSP assay are listed in Table S10, Supporting Information.

For Bisulfite Genomic Sequencing, primers were designed with MethPrimer 2.0 tools online (Table S10, Supporting Information). After PCR amplification was performed from deaminated DNA, products were gel‐purified and connected to T vector. Ten clones were randomly selected for sequencing and analysis by the use of NCBI Nucleotide BLAST.

### Co‐Immunoprecipitation

For co‐IP analysis, the H929‐Ctrl and H929‐TP53^KO^ cell lines were used. All procedures followed the standard protocol previously reported.^[^ ^11^, ^14^ ^]^ Briefly, cells were lysed in lysis buffer for 40 min on ice. The lysates were incubated overnight at 4 °C on a rotator with 4 µg of polyclonal anti‐p53 and mouse IgG antibodies (Santa Cruz Biotechnology, CA, USA). 50 µL of protein A/G beads (Biolinkedin, Shanghai, China) were transferred to the protein‐antibody complexes, and immunoprecipitates were collected after 2 h incubation. Finally, the immunoprecipitates were resuspended in lithium dodecyl sulfate (LDS) sample buffer and heated for 10–12 min at 70 °C for analysis by LDS polyacrylamide gel‐electrophoresis, loading equal concentrations of protein from the original lysate, and western blotting with monoclonal antibodies against p53 (ABclonal, Shanghai, China), DNMT1, and DNMT3B (Proteintech).

### Statistical Analysis

Quantitative data are shown as means ± SD. Student's *t*‐test, ANOVA with Dunnett post‐hoc test, Chi‐square and Rank sum tests were used to analyze data. To analyze correlation of *NEK2* and *TP53* expression with disease progression, overall survival was measured using the Kaplan‐Meier method, and the log‐rank test was used for group comparison based on GraphPad Prism 7 software. Significance was set at *p* < 0.05.

## Conflict of Interest

The authors declare no conflict of interest.

## Author Contributions

X.F. and J.G. contributed equally to this work. W.Z., X.F., and J.G. conceived of and designed the experiments; X.F., J.G., Y.W., B.M., Y.Z., L.Q., R.L., and J.X. performed the experiments; W.Z., X.F., J.G., Z.Y., and Z.L. analyzed the data; X.L., G.A., G.R., L.Q., and J.Z. provided critical materials; W.Z., X.F., and J.G. wrote the manuscript and all authors edited the manuscript.

## Supporting information

Supporting Information

Supplemental Table 1

Supplemental Table 2

Supplemental Table 3

Supplemental Table 4

Supplemental Table 5

Supplemental Table 6

Supplemental Table 7

Supplemental Table 8

Supplemental Table 9

Supplemental Table 10

Supplemental Figure 2

Supplemental materials‐and‐methods 2

Supplemental materials‐and‐methods 3

Supplemental materials‐and‐methods 4

## Data Availability

The data that support the findings of this study are available in the supplementary material of this article.
