# Supplementary material for: Genetic Aberrations and Interaction of NEK2 and TP53 Accelerate Aggressiveness of Multiple Myeloma
Source: Adv Sci (Weinh). 2022 Jan 27;9(9):2104491. doi: 10.1002/advs.202104491 (PMC8948659; doi:10.1002/advs.202104491)
Supplement: Supplementary file 1 — Supporting Information [file ADVS-9-2104491-s007.pdf]

ADVANCED SCIENCE Open Access

Supporting Information

for *Adv. Sci.*, DOI 10.1002/advs.202104491

Genetic Aberrations and Interaction of *NEK2* and *TP53* Accelerate Aggressiveness of Multiple Myeloma

*Xiangling Feng, Jiaojiao Guo, Gang An, Yangbowen Wu, Zhenhao Liu, Bin Meng, Nihan He, Xinying Zhao, Shilian Chen, Yinghong Zhu, Jiliang Xia, Xin Li, Zhiyong Yu, Ruixuan Li, Guofeng Ren, Jihua Chen, Minghua Wu, Yanjuan He, Lugui Qiu, Jiaxi Zhou and Wen Zhou**

ADVANCED SCIENCE
Open Access

Supporting Information

for *Adv. Sci.,* DOI: 10.1002/advs.202104491

Genetic Aberrations and Interaction of *NEK2* and *TP53* Accelerate Aggressiveness of Multiple Myeloma

Xiangling Feng, Jiaojiao Guo*, Gang An, Yangbowen Wu, Zhenhao Liu, Bin Meng, Nihan He, Xinying Zhao, Shilian Chen, Yinghong Zhu, Jiliang Xia, Xin Li, Zhiyong Yu, Ruixuan Li, Guofeng Ren, Jihua Chen, Minghua Wu, Yanjuan He, Lugui Qiu, Jiaxi Zhou, Wen Zhou*

Supporting information Figures

Genetic Aberrations and Interaction of *NEK2* and *TP53* Accelerate Aggressiveness of Multiple Myeloma

Xiangling Feng*, Jiaojiao Guo*, Gang An, Yangbowen Wu, Zhenhao Liu, Bin Meng, Nihan He, Xinying Zhao, Shilian Chen, Yinghong Zhu, Jiliang Xia, Xin Li, Zhiyong Yu, Ruixuan Li, Guofeng Ren, Jihua Chen, Minghua Wu, Yanjuan He, Lugui Qiu, Jiaxi Zhou, Wen Zhou#

Inventory of Supplemental Information

Supplemental Data

Figure S1, related to Figure 1.

Figure S2, related to Figure 2.

Figure S3, related to Figure 3.

Figure S4, related to Figure 4.

Figure S5, related to Figure 5.

Figure S6, related to Figure 6.

Figure S7, related to Figure 7.

Figure S8, related to Figure 8.

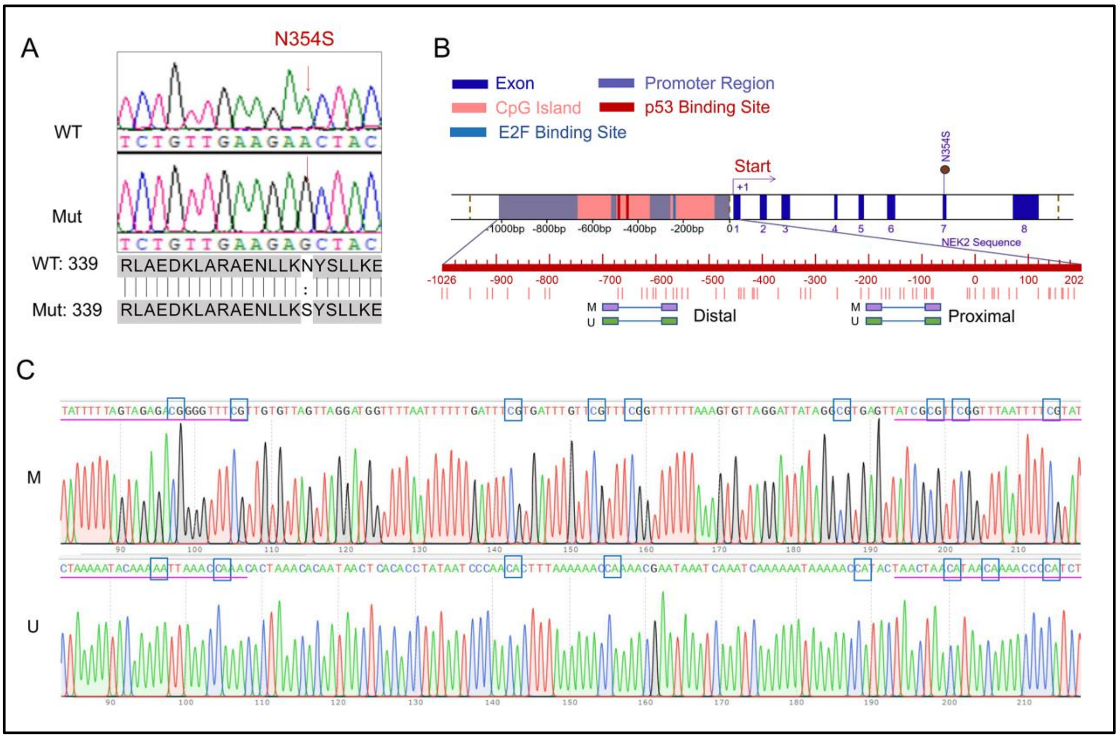

Figure S1, related to Figure 1. DNA status of *NEK2* in MM cell lines

(A) Representative results of DNA sequencing analysis for *NEK2* gene mutation. (B) Schematic depiction of *NEK2* DNA sequence including the hotspot mutation of *NEK2* enriched in the U266 cell line (N354S, marked with a red circle), predicted CpG islands in the *NEK2* promoter ranging from -1017bp~+212bp and the position of primers designed for MSP. Positions of the primers used for MSP analysis along with CpG sites of *NEK2* gene. (C) Sequencing analysis of methylated and unmethylated MSP products using the distal primers. All cytosines were converted to thymines except for those in CpG dinucleotides. The underlines show the MSP primers. The boxes show the CpG dinucleotides.

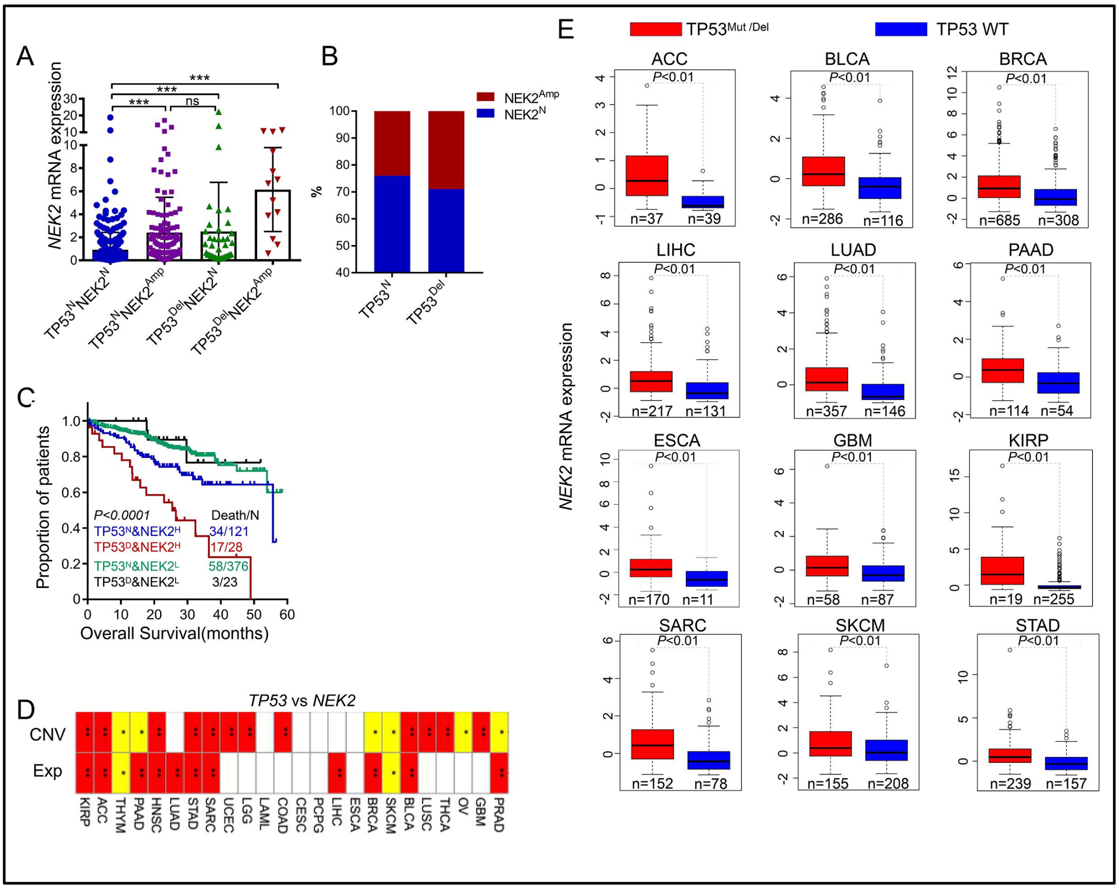

Figure S2, related to Figure 2. High *NEK2* expression in MM patients with *TP53* deletion/mutation

(A) Correlations between the amplification of *NEK2* copy numbers (n=13) and *NEK2* mRNA expression in MM patients with *TP53* deletion (n=51). (B) Proportion of patients with normal ($NEK2^{N}$) or amplified ($NEK2^{Amp}$) *NEK2* copy numbers among MM patients with or without *TP53* deletion. (C) Kaplan-Meier analyses of overall survival in MM patients with normal *TP53* ($TP53^{N}$) & low expression *NEK2* ($NEK2^{L}$), $TP53^{N}$ & high expression *NEK2* ($NEK2^{H}$), $TP53^{D}$ (deletion) & $NEK2^{L}$ and $TP53^{D}$ & $NEK2^{H}$ (n=548). (D) The correlation of *TP53* CNV and *NEK2* CNV or *NEK2* expression in multiple cancers by TCGA data analysis. (E) The correlation of genetic lesions (deletion and mutation) of *TP53* and *NEK2* expression in multiple cancers such as ACC, BLCA, BRCA, LIHC, LUAD, PAAD, ESCA, GBM, KIRP, SARC, SKCM, STAD by TCGA data analysis. Data are shown as mean ± SD. *$p < 0.05$, **$p < 0.01$, ***$p<0.001$.

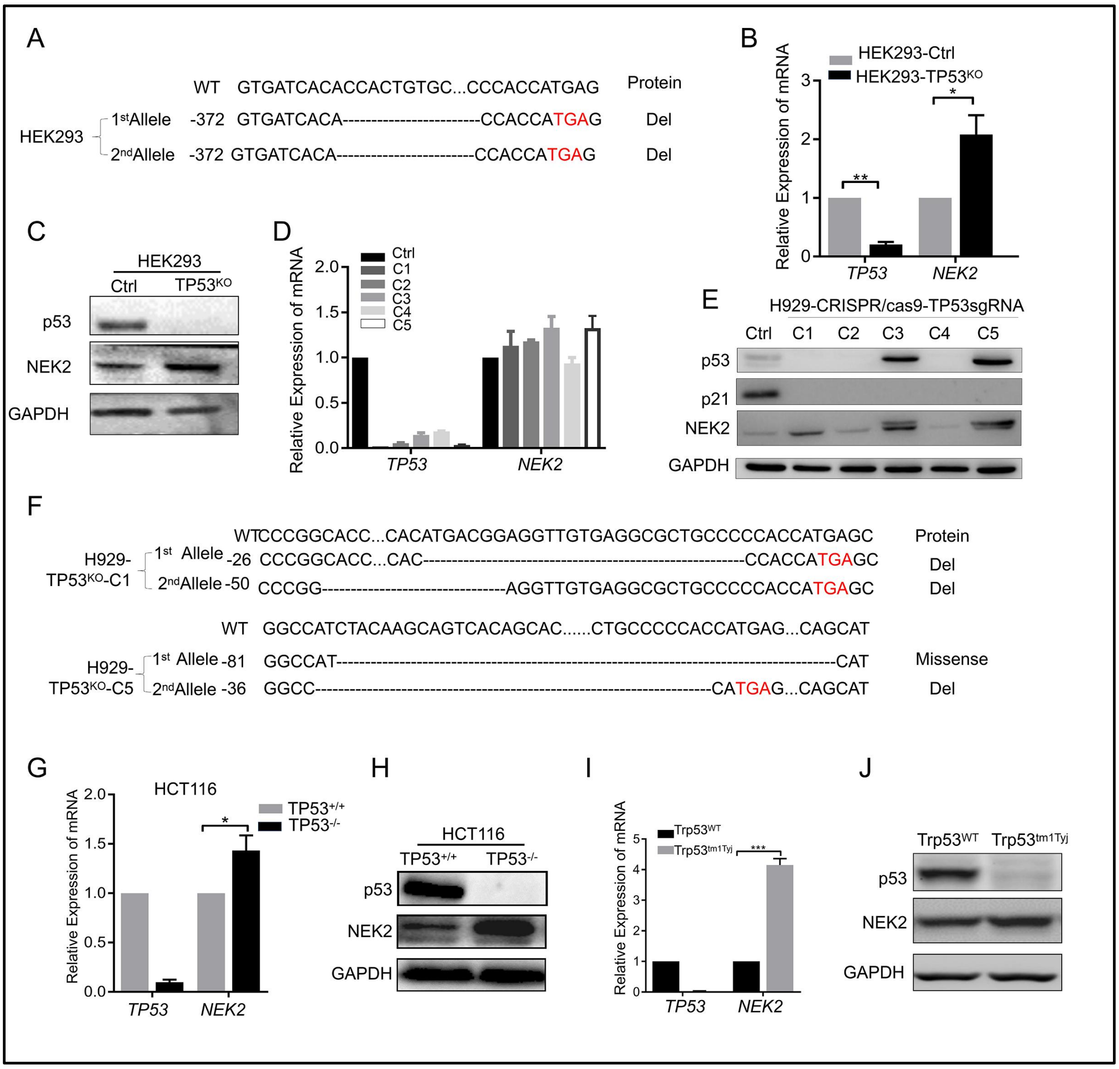

Figure S3, related to Figure 3. Establishment of TP53-deleted HEK293-TP53^{KO} and H929-TP53^{KO} cell lines via the CRISPR/Cas9 system

(A) *TP53* and *NEK2* mRNA levels in HEK293 cells with (HEK293-Ctrl) or without (HEK293-TP53KO) CRISPR-Cas9-mediated TP53 deletion, as determined with qPCR. (B) p53 and NEK2 protein levels in HEK293 cells with (HEK293-Ctrl) or without (HEK293-TP53^{KO}) CRISPR-Cas9-mediated TP53 deletion, as determined with immunoblotting. (C) Cell clones with TP53 deletions established from HEK293 cells edited by CRISPR-Cas9-TP53sgRNA were confirmed using DNA sequencing. (D&E) The mRNA and protein levels of *TP53* and *NEK2* in NCI-H929 cell clones with (NCI-H929-Ctrl) or without (NCI-H929-TP53^{KO} C1, C2, C3, C4, C5) *TP53* deletion, as determined with qPCR or immunoblotting, respectively. (F) Cell clones with *TP53* deletion established from NCI-H929-TP53^{KO} cell clones edited by CRISPR-Cas9-TP53sgRNA were confirmed using DNA sequencing. (G&H) Relative mRNA and protein levels of TP53 and NEK2 were detected with qPCR or immunoblotting, respectively, in paired TP53$^{WT/WT}$ and TP53$^{-/-}$ HCT116 cell lines. (I&J) Relative mRNA and protein levels of *TP53* and *NEK2* were detected with qPCR or immunoblotting, respectively, in TP53$^{+/+}$ and Trp53^{tm1Tyj} TP53 knockout mice.Data are shown as mean ± SD. *$p < 0.05$, **$p < 0.01$, ***$p<0.001$.

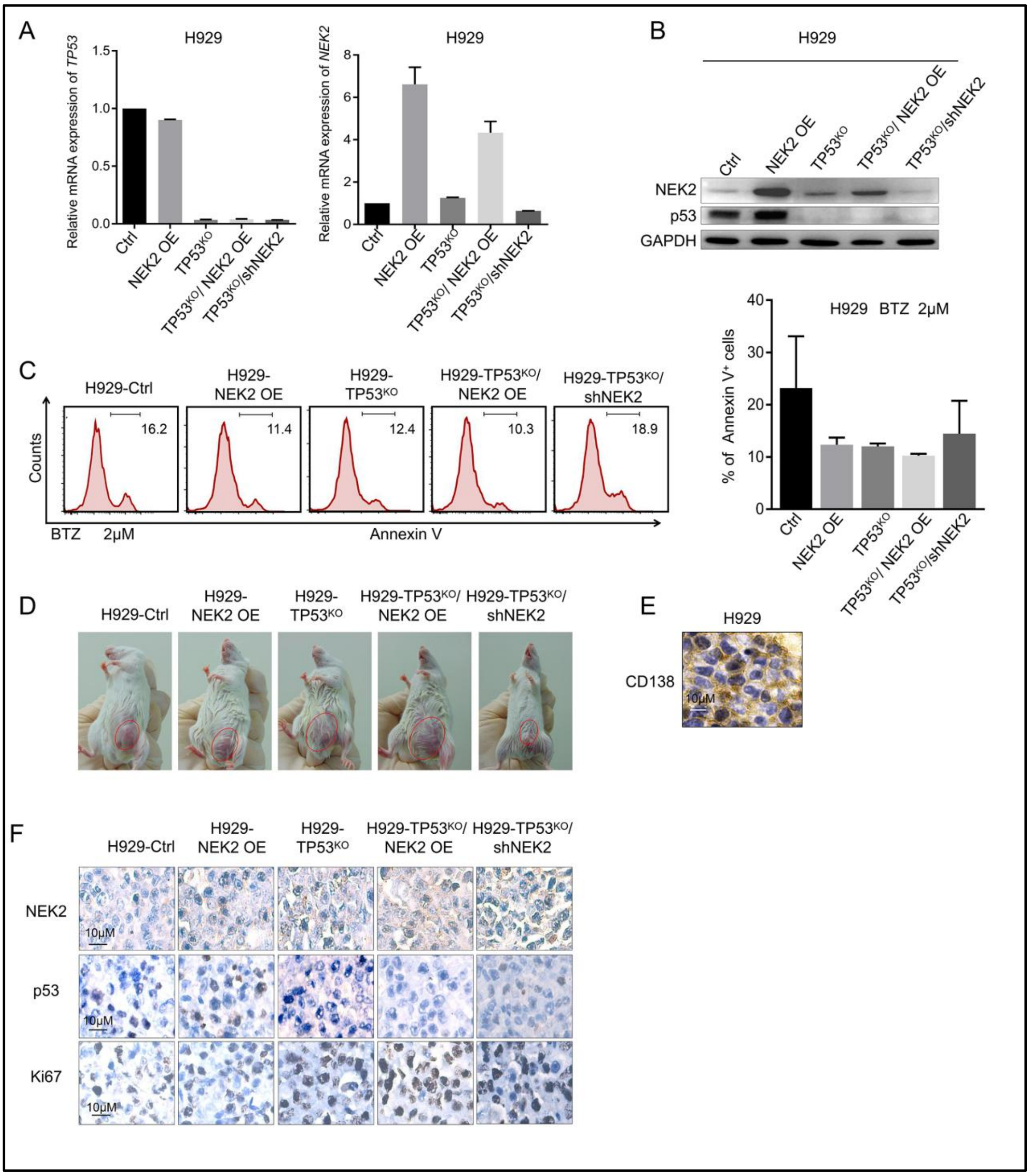

Figure S4, related to Figure 4. Dual defects in NEK2 and p53 enhance cell growth, apoptosis in MM

(A) Relative mRNA levels of *TP53* and *NEK2* were detected with qPCR in H929-Ctrl, H929-TP53^{KO}, H929-NEK2 OE, H929-TP53^{KO}/NEK2 OE and H929-TP53^{KO}/shNEK2 cells. (B) Relative protein levels of p53 and NEK2 were detected with immunoblotting in these five cell groups. (C) Representative images for detection and statistical analysis of the percentage of apoptotic cells in H929-Ctrl, H929-TP53^{KO}, H929-NEK2 OE, H929-TP53^{KO}/NEK2 OE and H929-TP53^{KO}/shNEK2 cells treated with 2 nM BTZ for 48 h. (D) Representative images of B-NDG mice with tumor xenografts from subcutaneous injection of H929-Ctrl, H929-TP53^{KO}, H929-NEK2 OE, H929-TP53^{KO}/NEK2 OE or H929-TP53^{KO}/shNEK2 cells into the right abdomen (six mice measured for each group). (E) Representative image for IHC detection of CD138 protein in the tumor nodules derived from B-NDG mice injected subcutaneously with H929-Ctrl, H929-TP53^{KO}, H929-NEK2 OE, H929-TP53^{KO}/NEK2 OE or H929-TP53^{KO}/shNEK2 cells. (F)

Representative images for IHC detection of p53 and NEK2 protein in the tumor nodules derived from B-NDG mice injected subcutaneously with H929-Ctrl, H929-TP53^{KO}, H929-NEK2 OE, H929-TP53^{KO}/NEK2 OE and H929-TP53KO/shNEK2 cells. Data are shown as mean ± SD.

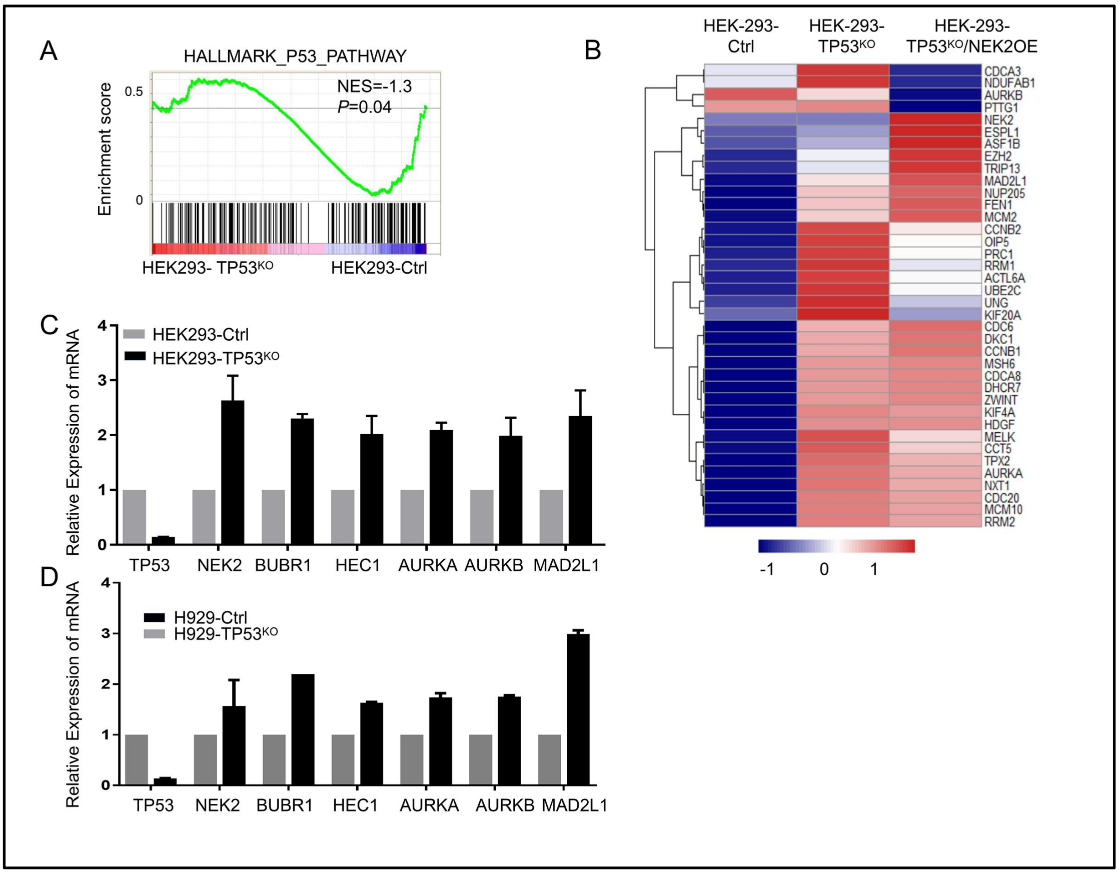

Figure S5, related to Figure 5. p53 deletion increased *NEK2* expression by regulating of genes involved in CIN and inducing *NEK2* amplification

(A) GSEA of p53 pathway-related genes from differentially expressed genes between HEK293-Ctrl and HEK293-TP53KO cells. (B) Heatmap of the ratios of the signal intensities of differential CIN genes in HEK293-Ctrl, HEK293-TP53^{KO} and HEK293-TP53^{KO}/NEK2 OE cells. (C&D) Relative mRNA levels of CIN genes were detected in HEK293-Ctrl and HEK293-TP53^{KO} (C) and H929-Ctrl and H929-TP53^{KO} (D) cells by qPCR. Data are shown as mean ± SD.

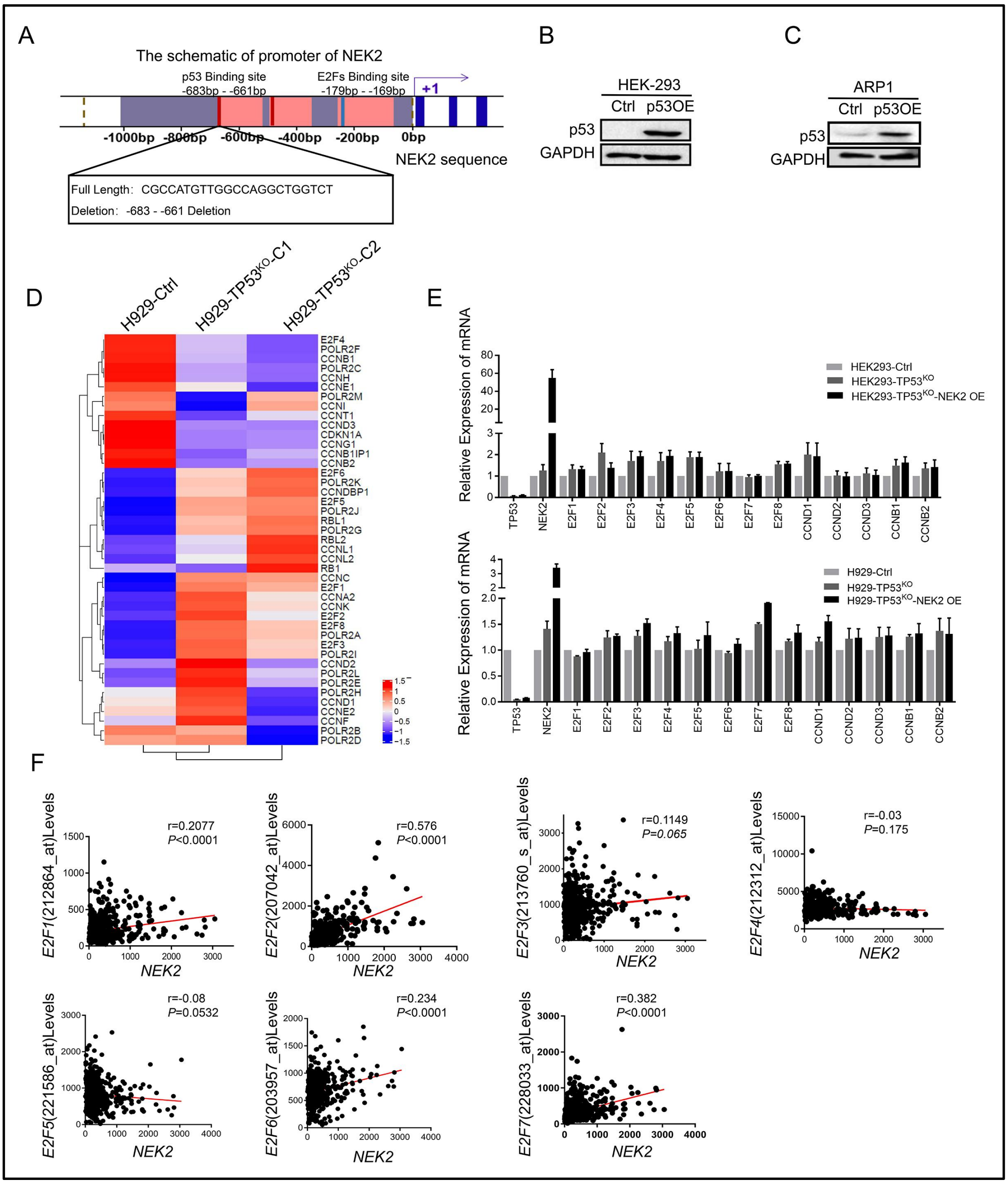

Figure S6, related to Figure 6. TP53 deletion enhances *NEK2* expression up-regulation of E2F8

(A) Schematic depiction of p53 and E2F8 binding sites in the *NEK2* promoter. (B&C) p53 protein levels were detected with immunoblotting in HEK293 (B) and ARP1 (C) cells. (D) Heatmap of the ratios of the signal intensities of differential cyclin-related genes in H929-Ctrl, H929-TP53^{KO} and H929-TP53^{KO}/NEK2 OE cells. (E) Relative mRNA levels of *E2F* and cyclin family genes were detected in HEK293-Ctrl, HEK293-TP53^{KO} and HEK293-TP53^{KO}/NEK2 OE cells (upper) and H929-Ctrl, H929-TP53^{KO} and H929-TP53^{KO}/NEK2 OE cells (lower) by qPCR. (F) The correlation between *NEK2* expression and expression of *E2F1*, *E2F2*, *E2F3*, *E2F4*, *E2F5*, *E2F6* and *E2F7* in MM patients based on GEP database (GSE2658, n=559). Data are shown as mean ± SD.

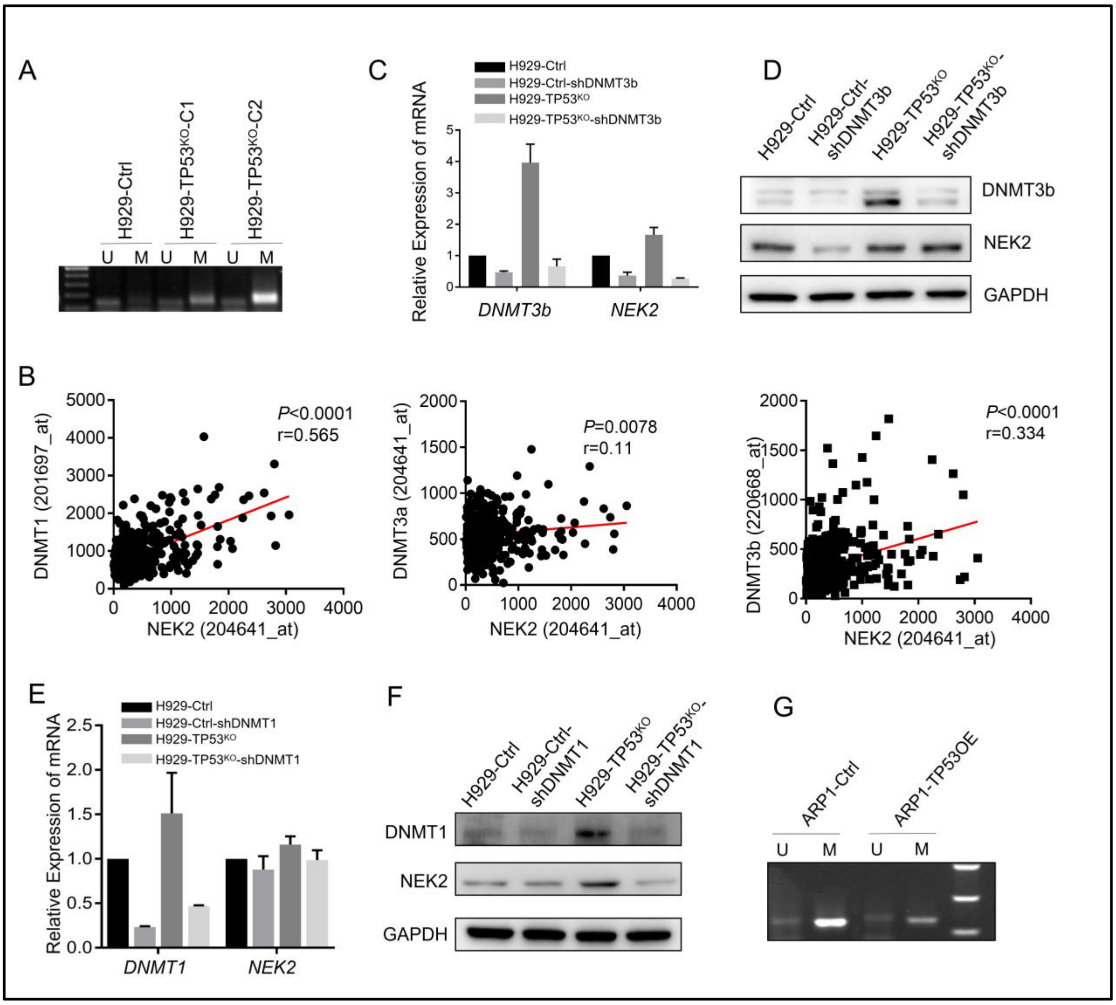

Figure S7, related to Figure 7. TP53 suppresses *NEK2* expression via downregulation of DNMTs

(A) The methylation status of the *NEK2* promoter's distal CpG islands in H929 cells with or without p53 using MSP. (B) The correlation between *NEK2* expression and expression of *DNMT1*, *DNMT3a* and *DNMT3b* in MM patients based on GEP database (GSE2658, n=559). (C&D) The mRNA and protein levels of *NEK2* after shRNA-mediated DNMT3b knockdown including target primer2 in wild type p53 and p53-deleted NCI-H929 cells, detected by qPCR and immunoblotting.(E&F) The mRNA and protein levels of *NEK2* after shRNA-mediated DNMT1 knockdown in WT p53 and p53-deleted NCI-H929 cells, detected by immunoblotting. (G) The methylation status of the *NEK2* promoter's distal CpG islands in ARP1 cells with or without p53, detected using MSP. Data are shown as mean ± SD.

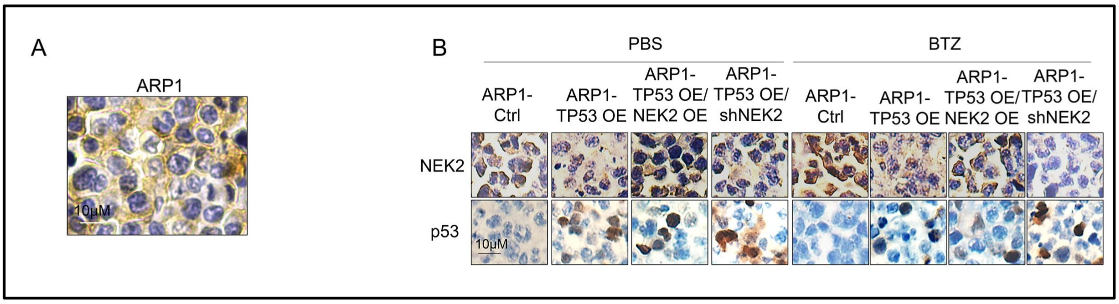

Figure S8, related to Figure 8. TP53 overexpression synergistically interacts with NEK2 suppression to promote tumor formation and reduce BTZ sensitivity

(A) Representative image for IHC detection of CD138 protein in the tumor nodules derived from B-NDG mice injected subcutaneously with ARP1-Ctrl, ARP1-TP53 OE, ARP1-TP53 OE/NEK2 OE or ARP1-TP53 OE/shNEK2 cells. (B) Representative images for IHC detection of p53 and NEK2 protein in the tumor nodules derived from B-NDG mice injected subcutaneously with ARP1-Ctrl, ARP1-TP53 OE, ARP1-TP53 OE/NEK2 OE or ARP1-TP53 OE/shNEK2 cells.
