# Supplementary material for: Genetic Aberrations and Interaction of NEK2 and TP53 Accelerate Aggressiveness of Multiple Myeloma
Source: Adv Sci (Weinh). 2022 Jan 27;9(9):2104491. doi: 10.1002/advs.202104491 (PMC8948659; doi:10.1002/advs.202104491)
Supplement: Supplementary file 2 — Supplemental Table 1 [file ADVS-9-2104491-s005.docx]

**Supplemental Table 1: related to Figure 1 and Figure 2.**

**Percentage of *NEK2* copy numbers in MM patients and MM cell lines**

| **Sample ID** | **Disease processes** | **Percentage of copy numbers** | |
| --- | --- | --- | --- |
|  |  | **3 copies** | **>3copies** |
| H1 | HD | 3.26% | 0.00% |
| H18 | HD | 1.72% | 0.00% |
| H24 | HD | 1.35% | 0.00% |
| H25 | HD | 2.90% | 0.00% |
| P2 | AD | 32.43% | 9.46% |
| P3 | AD | 33.96% | 4.72% |
| P4 | AD | 1.35% | 0.00% |
| P5 | AD | 16.16% | 3.03% |
| P6 | AD | 22.22% | 0.00% |
| P7 | AD | 9.38% | 0.00% |
| P8 | AD | 11.22% | 1.02% |
| P9 | AD | 20.69% | 0.00% |
| P10 | AD | 29.90% | 1.03% |
| P12 | AD | 13.27% | 0.00% |
| P15 | AD | 4.49% | 2.25% |
| P16 | AD | 6.67% | 5.00% |
| P17 | AD | 2.00% | 0.00% |
| P19 | AD | 12.20% | 6.10% |
| P20 | AD | 3.57% | 0.00% |
| P22 | AD | 10.26% | 1.28% |
| P23 | AD | 5.95% | 0.00% |
| P11 | RD | 23.33% | 1.11% |
| P13 | RD | 32.08% | 24.53% |
| P14 | RD | 36.36% | 6.06% |
| P21 | RD | 26.47% | 7.84% |
| P24 | AD-TP53^WT^ | 11.65% | 0.97% |
| P25 | AD-TP53^WT^ | 11.11% | 0.00% |
| P26 | AD-TP53^WT^ | 3.85% | 0.00% |
| P27 | AD-TP53^WT^ | 9.64% | 0.00% |
| P28 | AD-TP53^WT^ | 8.57% | 0.95% |
| P29 | AD-TP53^WT^ | 4.88% | 0.00% |
| P30 | AD-TP53^WT^ | 7.84% | 0.00% |
| P31 | AD-TP53^Del^(87%) | 25.86% | 18.97% |
| P32 | AD-TP53^Del^(92%) | 31.82% | 8.18% |
| P33 | AD-TP53^Del^(55%) | 44.44% | 3.70% |
| P34 | AD-TP53^Del^(94%) | 35.58% | 6.73% |
| NCI-H929 | MM cell line | 13.19% | 4.40% |
| MM.1s | MM cell line | 27.37% | 4.21% |
| MM.1R | MM cell line | 19.42% | 3.88% |
| 8226 | MM cell line | 34.72% | 20.83% |
| U266 | MM cell line | 4.88% | 18.29% |
| ARP1 | MM cell line | 39.62% | 11.32% |
| KMS11 | MM cell line | 46.32% | 33.68% |
| OCI-My5 | MM cell line | 33.67% | 18.37% |
