# Supplementary material for: Genetic Aberrations and Interaction of NEK2 and TP53 Accelerate Aggressiveness of Multiple Myeloma
Source: Adv Sci (Weinh). 2022 Jan 27;9(9):2104491. doi: 10.1002/advs.202104491 (PMC8948659; doi:10.1002/advs.202104491)
Supplement: Supplementary file 3 — Supplemental Table 2 [file ADVS-9-2104491-s004.docx]

**Supplemental Table 2: related to Figure 1.**

**Genetic and Epigenetic Changes of *NEK2* gene in MM cell lines**

| **Cell lines** | **Amplification** | **Mutation** | **Methylation** |
| --- | --- | --- | --- |
| NCI-H929 | N | N | Y |
| MM.1s | N | N | Y |
| MM.1R | N | N | Y |
| 8226 | Y | N | Y |
| U266 | N | Y | Y |
| ARP1 | Y | N | Y |
| KMS11 | Y | N | Y |
| OCI-My5 | N | N | Y |
