# Supplementary material for: Genetic Aberrations and Interaction of NEK2 and TP53 Accelerate Aggressiveness of Multiple Myeloma
Source: Adv Sci (Weinh). 2022 Jan 27;9(9):2104491. doi: 10.1002/advs.202104491 (PMC8948659; doi:10.1002/advs.202104491)
Supplement: Supplementary file 11 — Supplemental Table 10 [file ADVS-9-2104491-s006.docx]

Supplemental Table 10: related to materials and methods.

Summary for primer sequences

| The sgRNA guide sequences and the genotyping primers | | |
| --- | --- | --- |
| TP53(Target 1) | Forward | CTACAGTACTCCCCTGCCCT |
|  | Reverse | TAAGCAGCAGGAGAAAGCC |
| TP53(Target 1) | Forward | GAGGTGCTTACGCATGTTTG |
|  | Reverse | TGCACATCTCATGGGGTTAT |
| TP53(Target 1) | Forward | CCTTGGCTTTTGAAAATAAGCTCC |
|  | Reverse | ATTTACTTTGCACATCTCATGGGG |
| TP53(Target 1) | Forward | AGTCCTCTCTTTGCTGGCT |
|  | Reverse | TCTGCCTGTCTTCCAGATA |
| The primers used in the vector construction | | |
| NEK2-ORF | Forward | ATATAAACGGGGCCCAAGGCA |
|  | Reverse | GCTACAGGGAAACTGAAGAATTC |
| TP53-ORF | Forward | ACCTCCATAGAAGATTCTAGAATGGAGGAGCCGCAGTCAGA |
|  | Reverse | GATCGCAGATCCTTCGCGGCCGCTCAGTCTGAGTCAGGCCCTT |
| E2F8-ORF | Forward | GCTCTAGAGCAtggagaacgaaaaggaaaatctc |
|  | Reverse | CGGGATCCCGttaatggacatcctctgttgag |
| NEK2-  shRNA | sequences | GAT CCC CGC TGT AGT GTT GAA TAC TTG GTT CAA GAG ACC AAG TAT TCA ACA CTA CAG CTT TTT |
| E2F8-  shRNA | sequences | CCGGGCCGCAAAGACAAGTCTTTAACTCGAGTTAAAGACTTGTCTTTGCGGCTTTTT |
| DNMT1-shRNA | sequences | CCGGGCAGGCGGCTCAAAGATTTGCTATGGACACAAATCTTTGAGCCGCCTGCTTTTT |
| DNMT3b-shRNA 1 | sequences | CCGGTTGGCATTAGAATATCAGAGCCTCGAGGCTCTGATATTCTAATGCCAATTTTT |
| DNMT3b-shRNA 2 | sequences | CCGGAATTGCTGGGTACAACTTGGGCTCGAGCCCAAGTTGTACCCAGCAATTTTTTT |
|  | | |
|  | | |
| The primers used in the luciferase assay | | |
| pGL3-NEK2 promoter full length | Forward | GGGGTACCCCTGGTGTGTGGGTGCGTAAAAG |
|  | Reverse | CCCAAGCTTGGGGGCCCTGATCTCGCAGTC |
| pGL3-NEK2 promoter TP53 binding site mutation | Forward | GTAGAGACGGGGTTTCGTGAACTCTTGGCCTCG  AGC |
|  | Reverse | GCTCGAGGCCAAGAGTTCACGAAACCCCGTCTC  TAC |
| The primers used in the ChIP-qPCR assay | | |
| GAPDH | Forward | TACTAGCGGTTTTACGGGCG |
|  | Reverse | TCGAACAGGAGGAGCAGAGAGCGA |
| NEK2-TP53 | Forward | ACTCTTGGCCTCGAGCAATC |
|  | Reverse | GGGTCACGTTGAGGATATGCT |
| p21 | Forward | AGCAGGCTGTGGCTCTGATT |
|  | Reverse | CAAAATAGCCACCAGCCTCCTCT |
| NEK2-E2F8 | Forward | TTGGCGATCTCTATCAGAGGG |
|  | Reverse | AAAGTGTCACTAGGCAACCGC |
| The primers used in the RT-qPCR assay | | |
| GAPDH | Forward | TCGGAGTCAACGGATTTGGT |
|  | Reverse | TGGAATTTGCCATGGGTGGA |
| TP53 | Forward | GCTCAGATAGCGATGGTCTGG |
|  | Reverse | CGCCCATGCAGGAACTGTTA |
| NEK2 | Forward | CCAGCCCTGTATTGAGTC |
|  | Reverse | ACTTCCGTTCCTTTAGCA |
| BUBR1 | Forward | CTTAGGGTGCAGCTGGATGT |
|  | Reverse | ACCCATCCCAGAAGACCTGTA |
| HEC1 | Forward | TGCCCCTCATACGAACTTCC |
|  | Reverse | AGCCAAACTAAGGCTGCCAC |
| ARUKA | Forward | TTGGGTGGTCAGTACATGCT |
|  | Reverse | CCTGGCTCCCTCTGTTACAA |
| ARUKB | Forward | CATCCCAACATCCTGCGTCT |
|  | Reverse | ATGATCGTGGCTGTTCGCT |
| MAD2L1 | Forward | ACGGTGACATTTCTGCCACT |
|  | Reverse | TGGTCCCGACTCTTCCCATT |
| BLK | Forward | CAGAGGATGCCTGCTGGATT |
| CCNB1 | Reverse | TCAGACACCAGGATGTTGGC |
|  | Forward | GGGCTTGGAGAGGCAGTATC |
| CCNB2 | Reverse | AGTGTCTGAGCCAGTGCCAG |
|  | Forward | CACAGGATACACAGAGAATG |
| CCND1 | Reverse | CTTGATGGCGATGAATTTAG |
|  | Forward | CCTCTGTGCCACAGATG |
| CCND2 | Forward | CCTCCTACTTCCAGTGCGTG |
|  | Reverse | AGACAGGTAGCGATCCAGGT |
| CCND3 | Forward | CCTCCTACTTCCAGTGCGTG |
|  | Reverse | AGACAGGTAGCGATCCAGGT |
| E2F1 | Forward | GGATTTCACACCTTTTCCTGGAT |
|  | Reverse | CCTGGAAACTGACCATCAGTACCT |
| E2F2 | Forward | GAGTCAGAGGATGGGGTCCT |
|  | Reverse | TGCCTACCCACTGGATGTTG |
| E2F3 | Forward | AAGTGCCTGACTCAATAGAGAGCC |
|  | Reverse | AGTCTCTTCTGGACATAAGTAAACCTCA |
| E2F4 | Forward | GCAGACCCCACAGGTGTTTT |
|  | Reverse | GCTCCGAGCTCATGCACTCT |
| E2F5 | Forward | TTGCTTTAATGGTGATACACTTTTGG |
|  | Reverse | TCTGACCCATTTCTGGAATGG |
| E2F6 | Forward | GGCGAGGAAGTTACCCAGTC |
|  | Reverse | TTTTTGATGGCAGCAGGCCC |
| E2F7 | Forward | CCAGGCAGCCCAGACTAGAT |
|  | Reverse | ATTGGAGCTTTCGGGGCCAT |
| E2F8 | Forward | TGACGAAGTGGCAGAGGAAC |
|  | Reverse | GTACCTGTTTTTGGCGAGGC |
| GAPDH(m) | Forward | GGAGAAACCTGCCAAGTATGA |
|  | Reverse | TCCTCAGTGTAGCCCAAGA |
| TP53(m) | Forward | GCCATGGCCATCTACAAGAA |
|  | Reverse | AATTTCCTTCCACCCGGATAAG |
| NEK2(m) | Forward | CGAGAGCCTGATGAAGAACTAC |
|  | Reverse | GGCTGAGGATGGAAGATCAAG |
| The primers used in the MS-PCR assay | | |
| NEK2 M-Distal | Forward | TATTTTTAGTAGAGACGGGGTTTCGT |
|  | Reverse | ATACGAAAATTAAACCGAACGC |
| NEK2 U-Distal | Forward | TATTTTTAGTAGAGATGGGGTTTTGTT |
|  | Reverse | CTAAAAATACAAAAATTAAACCAAACACT |
| NEK2 M-Proximal | Forward | TATAGGTTAAAAGTAGACGTCGACG |
|  | Reverse | CAATCTATTAACAAACGATAAACCG |
| NEK2 U-Proximal | Forward | TTTTTTATAGGTTAAAAGTAGATGTTGATG |
|  | Reverse | CAATCTATTAACAAACAATAAACCACT |
