# Supplementary material for: Genetic Aberrations and Interaction of NEK2 and TP53 Accelerate Aggressiveness of Multiple Myeloma
Source: Adv Sci (Weinh). 2022 Jan 27;9(9):2104491. doi: 10.1002/advs.202104491 (PMC8948659; doi:10.1002/advs.202104491)
Supplement: Supplementary file 13 — Supplemental materials‐and‐methods 2 [file ADVS-9-2104491-s015.pdf]

ADVANCED SCIENCE
Open Access

Supporting Information

for *Adv. Sci.*, DOI 10.1002/advs.202104491

Genetic Aberrations and Interaction of *NEK2* and *TP53* Accelerate Aggressiveness of Multiple Myeloma

*Xiangling Feng, Jiaojiao Guo, Gang An, Yangbowen Wu, Zhenhao Liu, Bin Meng, Nihan He, Xinying Zhao, Shilian Chen, Yinghong Zhu, Jiliang Xia, Xin Li, Zhiyong Yu, Ruixuan Li, Guofeng Ren, Jihua Chen, Minghua Wu, Yanjuan He, Lugui Qiu, Jiaxi Zhou and Wen Zhou**

细胞 STR 分型检验报告

Report of Cell Line Identification

客户名称/ Applicant： 中南大学湘雅公共卫生学院
样本编号/ Sample No.： HEK293
待检测细胞系名称/Name of cell line: HEK293
样本数量及规格/ Sample Spec.： 细胞沉淀 1 个/ Cell precipitation
样本接收日期/ Sample Receive Date： 20200325
报告编号/ Report No： VC20200331002

1. **测试要求/**Service Description

鉴定该细胞样品是否存在交叉污染现象，并与 ATCC、DSMZ 数据库比对 DNA 分型数据确认来源。

Detection of human origin intra-species cross-contamination. Database search and analysis to identify cell origin of sample using two recognized repositories (ATCC, DSMZ).

2. **检材处理和检验方法/**Method and Procedure

取适量检材用莱枫痕量试剂盒提取 DNA，采用人类 STR 扩增荧光检测试剂盒进行复合 PCR 扩增，在 ABI 3730xl 型遗传分析仪上对 STR 位点和性别基因 Amelogenin 进行检测。

Cellular DNA is purified with lifefeng DNA kit. PCR is amplified with Human STR Identification Kit. PCR products are assayed with 3730xl DNA Analyzer (Applied Biosystems).

3. **检验结果/**STR Profiles

该细胞株的 STR 位点和 Amelogenin 位点的基因分型结果见附表 1，分型图谱见附图 1。

The STR profiles of the cell line sample are summarized in Table 1 and Figure 1.

4. **检验结论/**Result & Analysis

样本编号/Sample No.：HEK293

1) 用 GeneMapperID-X 1.4 software(ABI)对各 STR 位点进行基因型分析。该细胞 DNA 扩增后图谱清晰，分型结果良好。
   STR Typing profile is analyzed with GeneMapper ID-X 1.4 software (Applied Biosystems) (Table 1 and Figure 1).
2) 性别基因 Amelogenin：X。
3) 该株细胞 DNA 进行细胞 STR 分型结果显示（如图 1），未出现多等位基因。
   Normal peaks distribution was observed (Figure 1).
4) 该株细胞 DNA 分型在 DSMZ 数据库中找到与其细胞分型 94%匹配的细胞（HEK-293.2sus, DSMZ P/N: CRL-1573.3 等，如图 2,3）。
   94% matched cell line (HEK-293.2sus, DSMZ P/N: CRL-1573.3 etc.) is found in DSMZ data bank (Figure 2 and Figure 3).

审核人/Reviewed by：邓昌焕/Changhuan Deng

VivaCell Biosciences 上海龙田生物科技有限公司 检验专用章

表 1：样本 HEK293 的 STR 位点和 Amelogenin 位点的基因分型结果

Table 1:STR profiles of Sample

	**Sample** HEK293	**Source: DSMZ;** HEK-293.2sus,94% Match
**Marker**	**Allele**	**Allele**
D3S1358	15,17	
D5S818	8	8
D2S1338	19	
TPOX	11	11
CSF1PO	12	12
Penta D	9	
Indel		
AMEL	X	X
TH01	7,9.3	7,9.3
vWA	16,19	16,19
D7S820	11	11,12
D21S11	30.2	
Penta E	7,15	
D10S1248	14	
D8S1179	12,14	
D1S1656	15,17.3	
D18S51	17	
D12S391	19,21	
D6S1043	11	
D19S433	15,18	
D16S539	9,13	9,13
D13S317	12,14	12,14
FGA	23	

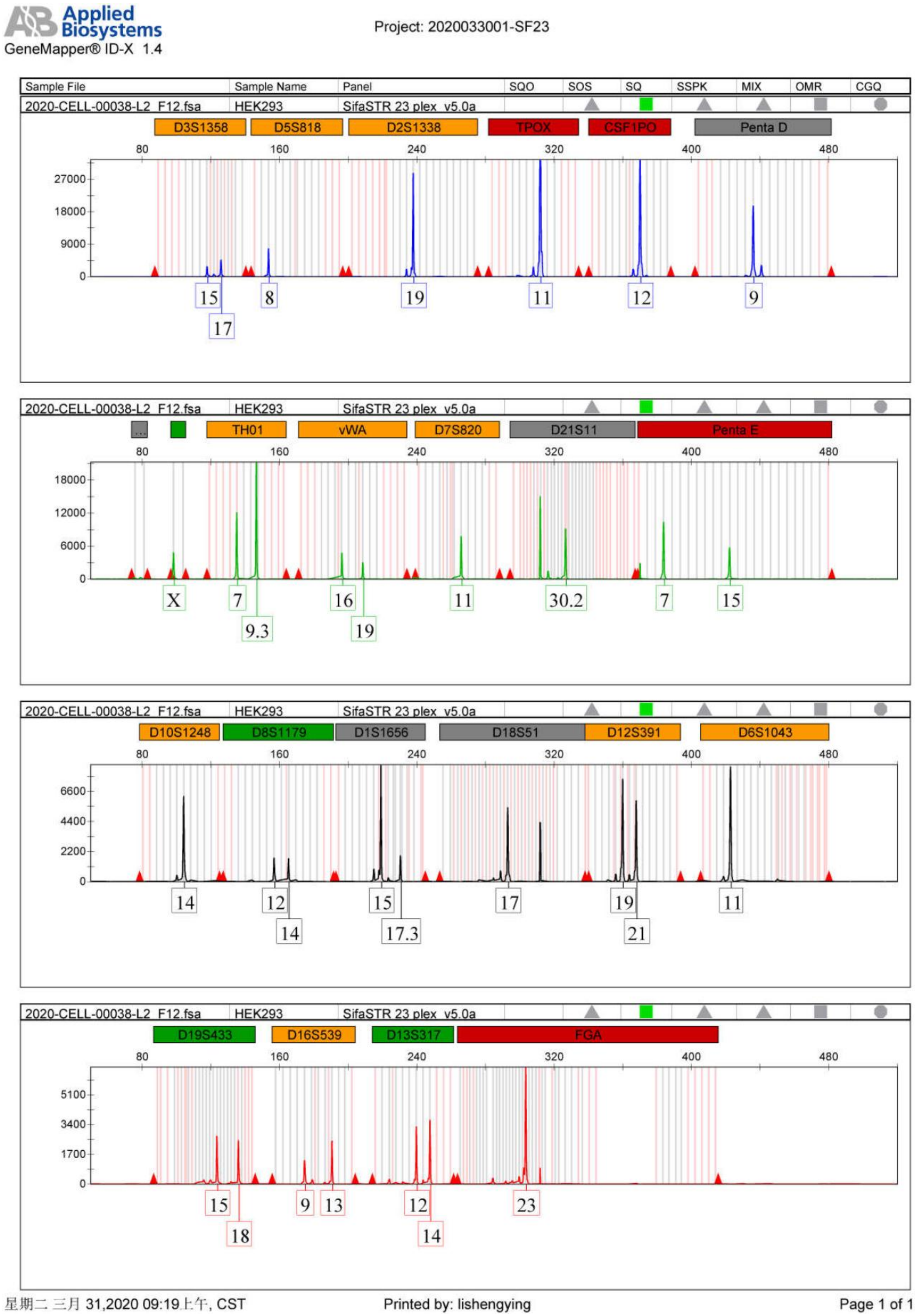

图 1/Figure 1：样本 HEK293 的 STR 位点和 Amelogenin 位点的基因分型图（STR profiles of Sample）

Add to Cart	%Match	ATCC® Number	Designation	D5S818	D13S317	D7S820	D16S539	vWA	TH01	AMEL	TPOX	CSF1PO
	93.0	CRL-2029	WSS-1 (WS-1)KidneyHuman	8	12,14	11,12	9,13	16,19	7,9.3	X	11	12
	93.0	CRL-2368	293/CHE-Fc Kidney,transfected; Human	8	12,14	11,12	9,13	16,19	7,9.3	X	11	12
	93.0	CRL-12006	ProPakA.6 Embryonic Kidney Human	8	12,14	11,12	9,13	16,19	7,9.3	X	11	12
	93.0	CRL-2794	GFPu-1Embryonic KidneyHuman	8	12,14	11,12	9,13	16,19	7,9.3	X	11	12
	93.0	CRL-1573.3	HEK-293.2susEmbryonic KidneyHuman	8	12,14	11,12	9,13	16,19	7,9.3	X	11	12
	93.0	CRL-3269	NaV 1.3 KIR 2.1Embryonic KidneyHuman	8	12,14	11,12	9,13	16,19	7,9.3	X	11	12
	92.0	CRL-12013	2A Embryonic Kidney Human	8,9	12,14	11	9	16,19	7,9.3	X	11	12
	87.0	CRL-3215	Phoenix GP Cells	8,9	12,14	11	9,13	16,19	7,9.3	X	11	11,12
	87.0	ACS-4500	HEK293T/17 SFSuspension Cells	8,9	12,14	11	9,13	16,19	7,9.3	X	11	11,12

图 2/Figure 2：样本 HEK293 与 ATCC 数据对比分析图（Sample Comparison to the ATCC STR Profile Database)

Result of STR matching analysis by your data.

- DSMZ Profile Database -

A graphical presentation is shown at the bottom of this page.

EV	Cell No.	Cell name	Locus names									Figures
			D5S818	D13S317	D7S820	D16S539	VWA	TH01	AM	TPOX	CSF1PO	
		Query (Your Cell)	*8,8*	*12,14*	*11,11*	*9,13*	*16,19*	*7,9.3*	*X,X*	*11,11*	*12,12*	
0.94(34/36)	CRL-12006	ProPakA.6	8,8	12,14	11,12	9,13	16,19	7,9.3	X,X	11,11	12,12	-
0.94(34/36)	CRL-1573.3	HEK-293.2sus	8,8	12,14	11,12	9,13	16,19	7,9.3	X,X	11,11	12,12	-
0.94(34/36)	CRL-2029	WSS-1 [WS-1]	8,8	12,14	11,12	9,13	16,19	7,9.3	X,X	11,11	12,12	-
0.94(34/36)	CRL-2368	293/CHE-Fc	8,8	12,14	11,12	9,13	16,19	7,9.3	X,X	11,11	12,12	-
0.94(34/36)	CRL-2782	293 EcR Shh [JHU-64]	8,8	12,14	11,12	9,13	16,19	7,9.3	X,X	11,11	12,12	-
0.94(34/36)	CRL-2794	GFPu-1	8,8	12,14	11,12	9,13	16,19	7,9.3	X,X	11,11	12,12	-
0.89(32/36)	635	293T	8,9	12,14	11,11	9,13	16,19	7,9.3	X,X	11,11	11,12	-
0.89(32/36)	ACS-4500	HEK 293T/17	8,9	12,14	11,11	9,13	16,19	7,9.3	X,X	11,11	11,12	-
0.89(32/36)	CRL-12013	2A	8,9	12,14	11,11	9,9	16,19	7,9.3	X,X	11,11	12,12	-
0.89(32/36)	RCB2202	293T	8,9	12,14	11,11	9,13	16,19	7,9.3	X,X	11,11	11,12	-

图 3/ Figure 3：样本 HEK293 与 DSMZ 数据对比分析图（Sample Comparison to the DSMZ STR Profile Database）
