# Supplementary material for: Genetic Aberrations and Interaction of NEK2 and TP53 Accelerate Aggressiveness of Multiple Myeloma
Source: Adv Sci (Weinh). 2022 Jan 27;9(9):2104491. doi: 10.1002/advs.202104491 (PMC8948659; doi:10.1002/advs.202104491)
Supplement: Supplementary file 14 — Supplemental materials‐and‐methods 3 [file ADVS-9-2104491-s012.docx]

**Supporting information 16: Construction of expression vector containing *TP53* point mutation**

In order to explore the effect of mutant p53 on *NEK2* expression, we chose four hotspot mutations in p53 in multiple tumors (Figure 1) and constructed expression vectors of p53 mutation. We used the pCDH vector containing the wild type *TP53* sequence as a template to obtain the mutant p53 expression vector by site-directed mutagenesis and confirmed by DNA sequencing (Figure 2).


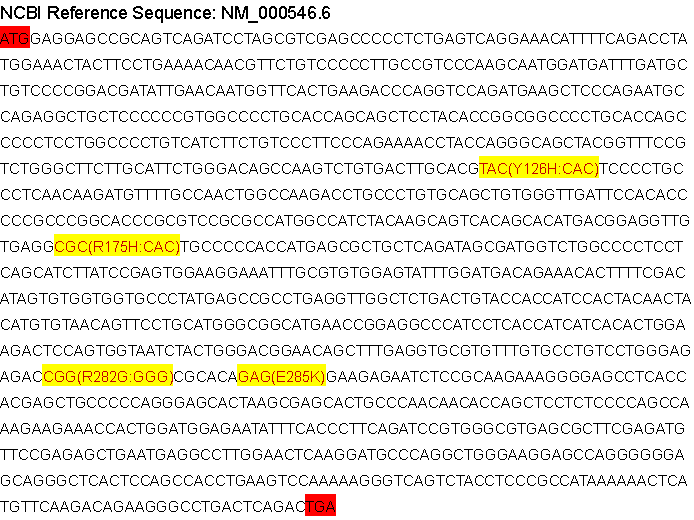


Figure 1. Schematic diagram of *TP53* mutation site


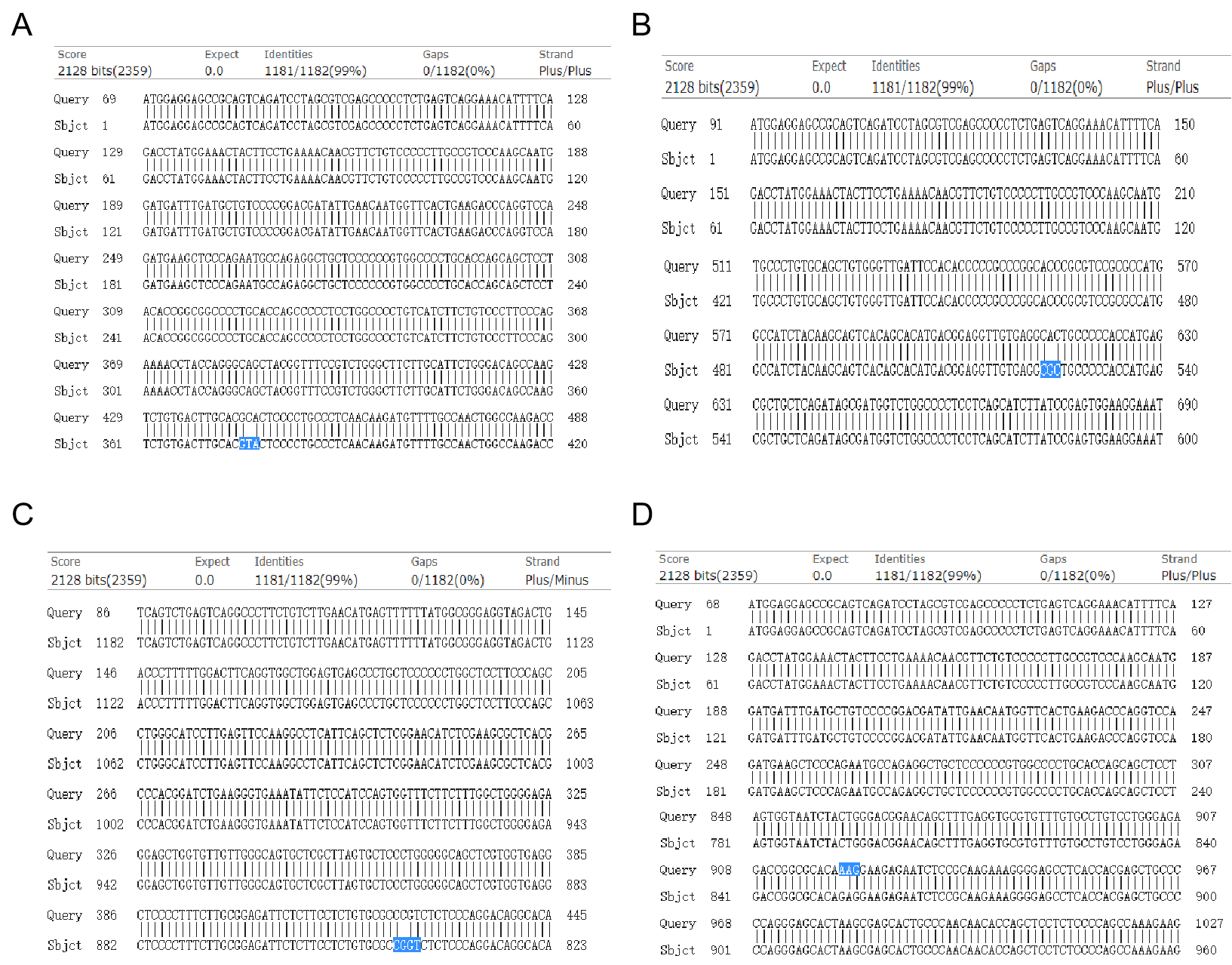


Figure 2. DNA sequencing confirms mutation sites in constructed mutant p53 expression vectors

1. Y126H changed in p53 expression vector. (B) R175H changed in p53 expression vector. (C) R282G changed in p53 expression vector. (D) E285K changed in p53 expression vector. All mutation sites are marked in blue.
