# Supplementary material for: Genetic Aberrations and Interaction of NEK2 and TP53 Accelerate Aggressiveness of Multiple Myeloma
Source: Adv Sci (Weinh). 2022 Jan 27;9(9):2104491. doi: 10.1002/advs.202104491 (PMC8948659; doi:10.1002/advs.202104491)
Supplement: Supplementary file 15 — Supplemental materials‐and‐methods 4 [file ADVS-9-2104491-s009.docx]

**Supporting information 6: Construction of H929 cells with CRISPR/Cas9-mediated *TP53* disruption**

qPCR and western blotting were used to confirm deletion of the *TP53* gene in selected NCI-H929 cell clones. In addition, PCR products amplified off genomic DNA separation and covering the *TP53* sgRNA target region were inserted into the T vector. Then, several bacterial colonies were selected for sequencing and compared with the wild type *TP53* DNA and protein sequence (NM_000546.6) (Figure 2) by Align Sequences Nucleotide BLAST. The individual reads are shown to indicate indels present in the respective CRISPR/Cas9-edited H929 clones (Figure 1). Figure 2 shows the predicted protein changes.


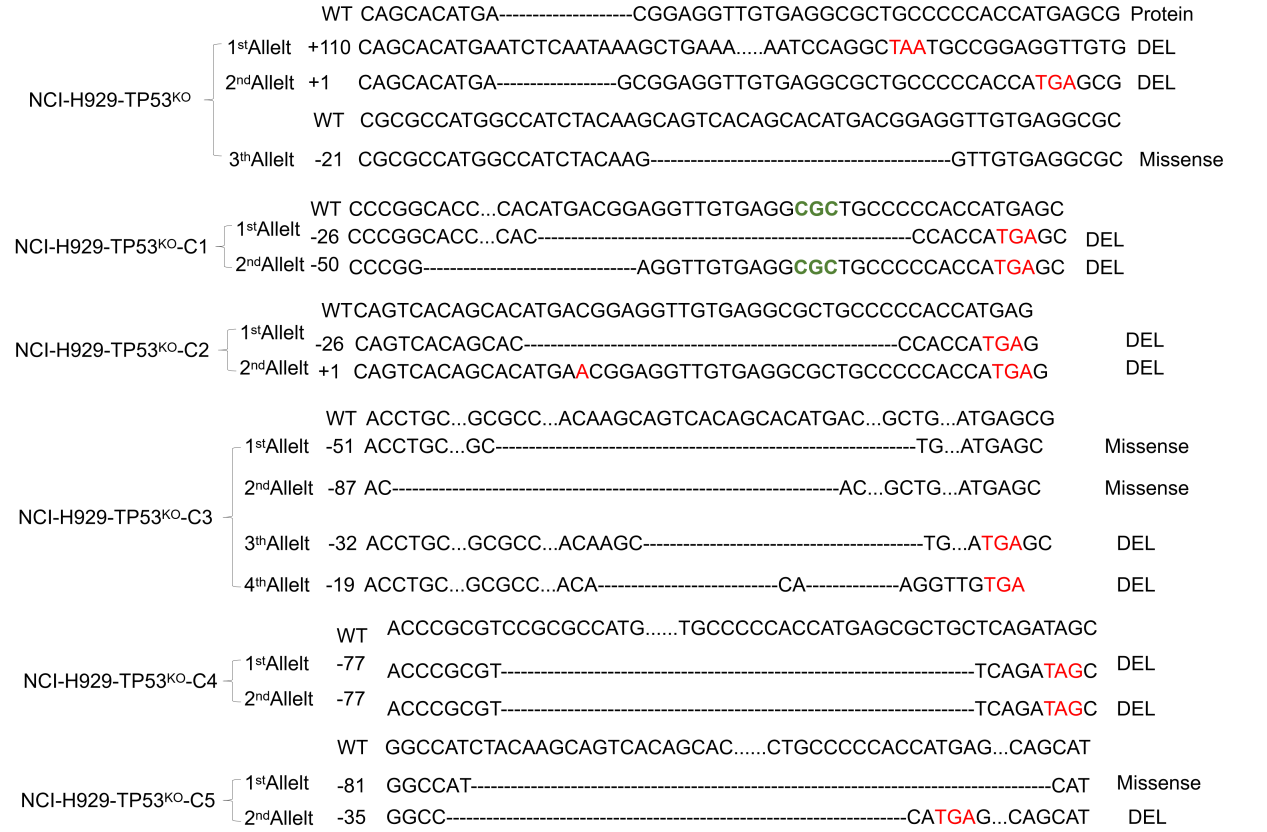


Figure 1. Sequences analysis of CRISPR-Cas9-TP53 sgRNA-mediated *TP53* indels in H929 cell clones


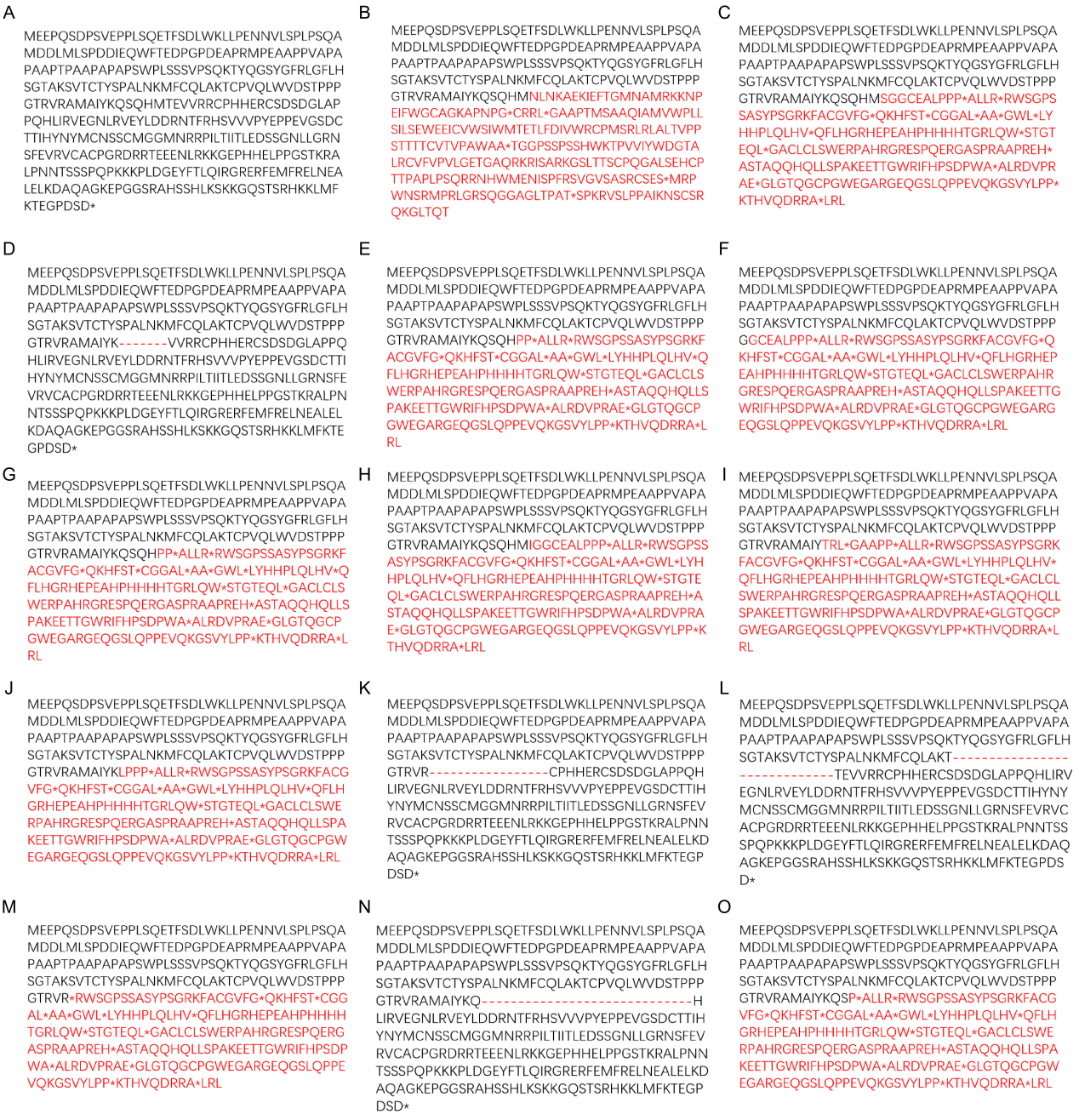


Figure 2. Analysis of predicted p53 protein sequences in individual cell clones edited by CRISPR/Cas9

1. : p53 protein sequence in the NCI-H929 cell line. (B) and (C): Early termination of p53 protein translation in H929-TP53^KO^ cells. (D): Seven missing amino acids of the p53 protein in H929-TP53^KO^ cells. (E) and (F): Early termination of translation in H929-TP53^KO^-C1 cells. (G) and (H) Early termination of p53 translation in H929-TP53^KO^-C2 cells. (I) and (J): Early termination of p53 translation in H929-TP53^KO^-C3 cells. (K): 17 missing amino acids of the p53 protein in H929-TP53^KO^-C3 cells. (L): 29 missing amino acids of the p53 protein in H929-TP53^KO^-C3 cells. (M): Early termination of p53 translation in H929-TP53^KO^-C4 cells. (N): 27 Missing amino acids of the p53 protein in H929-TP53^KO^-C5 cells. (O): Early termination of p53 translation in H929-TP53^KO^-C5.
